# Dissolved organic matter transformations in a freshwater rivermouth

**DOI:** 10.1007/s10533-022-01000-z

**Published:** 2023-03-02

**Authors:** Nolan J. T. Pearce, James H. Larson, Mary Anne Evans, Sean W. Bailey, Paul C. Frost, William F. James, Marguerite A. Xenopoulos

**Affiliations:** 1 Department of Biology, Trent University, Peterborough, ON Canada; 2 Upper Midwest Environmental Sciences Center, U.S. Geological Survey, La Crosse, WI USA; 3 Great Lakes Science Center, U.S. Geological Survey, Ann Arbor, MI USA; 4 Discovery Center, Center for Limnological Research and Rehabilitation, University of Wisconsin Stout, Menomonie, WI USA

**Keywords:** Carbon, Water column, Sediments, Nutrients, PARAFAC

## Abstract

River-to-lake transitional areas are biogeochemically active ecosystems that can alter the amount and composition of dissolved organic matter (DOM) as it moves through the aquatic continuum. However, few studies have directly measured carbon processing and assessed the carbon budget of freshwater rivermouths. We compiled measurements of dissolved organic carbon (DOC) and DOM in several water column (light and dark) and sediment incubation experiments conducted in the mouth of the Fox river (Fox rivermouth) upstream from Green Bay, Lake Michigan. Despite variation in the direction of DOC fluxes from sediments, we found that the Fox rivermouth was a net sink of DOC where water column DOC mineralization outweighed the release of DOC from sediments at the rivermouth scale. Although we found DOM composition also changed during our experiments, alterations in DOM optical properties were largely independent of the direction of sediment DOC fluxes. We found a consistent decrease in humic-like and fulvic-like terrestrial DOM and a consistent increase in the overall microbial composition of rivermouth DOM during our incubations. Moreover, greater ambient total dissolved phosphorus concentrations were positively associated with the consumption of terrestrial humic-like, microbial protein-like, and more recently derived DOM but had no effect on bulk DOC in the water column. Unexplained variation indicates that other environmental controls and water column processes affect the processing of DOM in this rivermouth. Nonetheless, the Fox rivermouth appears capable of substantial DOM transformation with implications for the composition of DOM entering Lake Michigan.

**Supplementary Information:**

The online version contains supplementary material available at 10.1007/s10533-022-01000-z.

## Introduction

The potential for inland waters to alter the flux of carbon from land to ocean is now widely accepted with recent estimates of about 70% of terrestrial carbon inputs outgassed along the aquatic continuum (Drake et al. 2018). Dissolved organic matter (DOM) is a major component of freshwater carbon pools and is actively transformed as it flows through inland waters (Battin et al. 2008; Tranvik et al. 2009). Despite this, estimates of the amount ofDOM produced and removed in transitional ecosystems such as rivermouths are often excluded from larger-scale carbon budgets leaving us with uncertainty in how they influence the downstream transport of these materials.

Rivermouths or river-estuary systems are biogeochemically important locations along the aquatic continuum (Larson et al. 2013; Xenopoulos et al. 2017). These transitional areas between lotic and lentic environments support high rates of aquatic metabolism as water residence time increases and nutrient availabilities remain elevated (Larson et al. 2013). Because of this distinct physiography, rivermouths provide the opportunity for metabolic processes to substantially alter the movement and composition of materials flowing downstream. Indeed, aquatic metabolism has been shown to alter the concentration and form of nutrients (i.e. nitrogen and phosphorus) transported from rivermouths to nearshore waters of the Laurentian Great Lakes (Klarer and Milie 1989; Conroy et al. 2017; Larson et al. 2019; Pearce et al. 2021) and marine environments (Fisher et al. 1988; Fong and Zedler 2000; Levin et al. 2001). However, few studies have investigated the biological and physical transformations of DOM in rivermouths (but see Larson et al. 2016).

Like lakes and reservoirs (e.g. Cole et al. 2007; Battin et al. 2009), rivermouths can also affect the fate of DOM through the flocculation and deposition of particulate organic matter, but it is not clear if organic matter mineralization through microbial activities and photochemical reactions would outweigh long-term sediment storage in these more turbulent, shallower, and often warmer waters. Moreover, DOM within pore water and at the sediment-water interface can interact with sediments and become temporarily removed from advective transport (Chen and Hur 2015). However, the stability of this sediment-bound organic matter may differ with DOM complexity and can be readily mobilized under changing redox conditions (Skoog and Arias-Esquivel 2009; Peter et al. 2016) and/or across steeper dissolved organic carbon (DOC) gradients at the sediment-water interface (Tao et al. 2000; Jin et al. 2008). Dynamic inflows and differential mixing of river and lake water within rivermouths may alter the chemical conditions of the overlying water column and result in more active organic matter transformations in sediment porewater.

As in rivers (e.g. Casas-Ruiz et al. 2017; Creed et al. 2015) and lakes (e.g. Larson et al., 2007; de Wit et al. 2018), water column and benthic processes in rivermouths are expected to affect both the production and consumption of organic materials and ultimately alter the amount (i.e. DOC concentration) and chemical composition of DOM transported downstream. However, rivermouths are often excluded from carbon and nutrient budgets as tributary inputs are typically measured at riverine gauges upstream from these more complex transitional areas. Research on freshwater rivermouths has shown differences in DOM composition and DOC concentrations from upstream rivers to downstream lakes (Larson et al. 2016), but the biogeochemical influence of rivermouths on the transport and transformation of these materials is largely unknown. The quantity and composition of DOM exported from inland waters is critical to the condition and functioning of recipient ecosystems (e.g. Xenopoulos et al. 2021). Moreover, fundamental knowledge on rivermouth processes is needed to refine our understanding of carbon movement across the aquatic continuum and quantify carbon budgets at larger scales (Cory and Kling, 2018). Our goal was therefore to describe the processing of DOM in a freshwater rivermouth by upscaling transformation rates from in situ water column bioassays and laboratory sediment incubations. A secondary goal was to determine the influence of dissolved nutrients (i.e. nitrogen and phosphorus) on temporal variation in volumetric water column DOM processing. We expected the net mineralization of DOC at the rivermouth scale as a result of water column processing and a connection of these rates to dissolved nutrients due to their influence on aquatic metabolism. Moreover, we predicted that DOM composition would change as a result of biological and physical processing in the rivermouth.

## Methods

### Study area

**Fig. 1:**
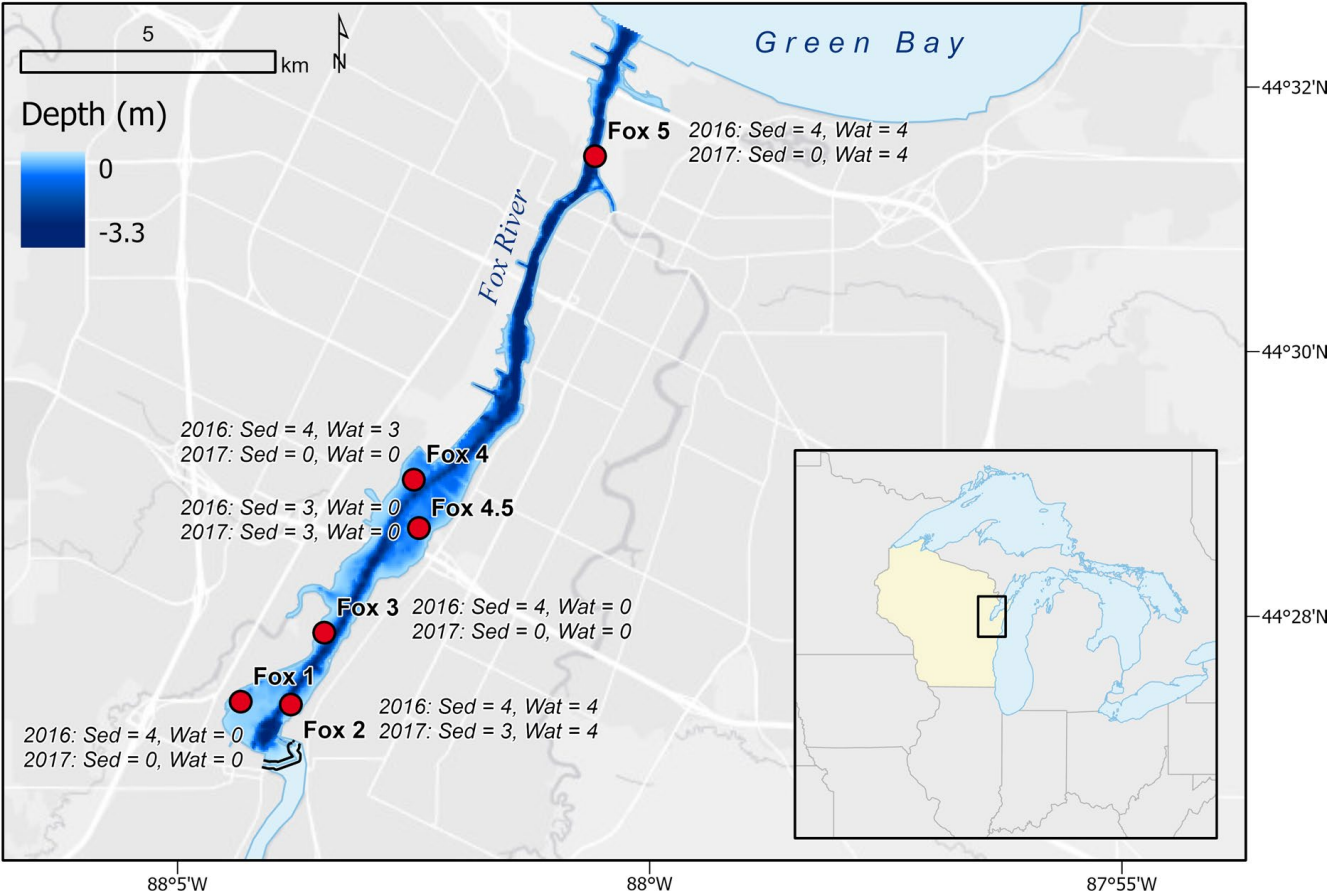
Location of study sites on the mouth of the Fox River downstream from the De Pere dam (black lines) in northeastern Wisconsin, USA. The number and type of incubation experiments (Sed = sediment; Wat = water column) are annotated beside site locations. Bathymetry (depth, meters) obtained from the National Oceanic and Atmospheric Administration, National Centers for Environmental Information. Service layer credits: Esri, HERE, Garmin, GeoTechnologies, Inc., USGS, EPA.

Water column and sediment incubation experiments occurred in the river-to-lake transition zone between the Fox River and Green Bay, Lake Michigan (Fig. 1). The Fox River is located in northeastern Wisconsin (USA) and drains an agricultural watershed that is heavily urbanized downstream from Lake Winnebago (Kreiling et al., 2020). The Fox River watershed is within the eastern temperate forest ecoregion (level II) and is characterized by a post-glacial physiography with a humid continental climate (Omernik and Griffith 2014). Six sites along the mouth of the Fox River (hereafter Fox rivermouth), located between the De Pere Dam and Green Bay, Lake Michigan, were used for sediment and water column incubation experiments in 2016 and 2017. Within each study year, sediment and water column incubation experiments were conducted in May/April, June, August, and September. Logistical constraints and compromised samples resulted in differences in the total number of sites sampled between study years (Fig. 1).

### Sediment incubations

Sediment core collection and incubation followed methods previously described in Larson et al. (2020). In brief, three replicate sediment cores were collected at each sampling location for incubation experiments. Sediment cores were collected with a gravity corer (Aquatic Research Instruments, Hope, Idaho; 20 cm height, 6.5 cm diameter) equipped with an acrylic liner. Collected sediment cores with overlying water were then sealed with stoppers and stored on ice (< 24 h) prior to laboratory incubation experiments. Rivermouth water was collected alongside sediment cores and was transported on ice to serve as the overlying water for the sediment incubation systems and to measure changes in DOC concentration and DOM composition. Sediment cores were incubated in the laboratory (University of Wisconsin-Stout) under aerobic conditions (typical of the Fox rivermouth) at ambient collection temperatures in darkened environmental chambers. Prior to incubation, overlying core water was siphoned off and the thickness of each core was adjusted to about 10 cm within the acrylic liner. Collected rivermouth water was filtered through a glass fiber filter (Gelman A/E glass fiber 1.0 μm) and 300 mL (~ 0.09 m depth) was carefully siphoned onto the intact core, while minimizing sediment disturbance and resuspension, to serve as overlying water. An initial sample of rivermouth water and periodic water samples (40 mL) were collected from each incubated core and were filtered through a pre-rinsed 0.22-µm polycarbonate membrane and stored in amber glass vials at 4^o^C prior to DOC and DOM analyses. The total duration of sediment incubations varied among sampling events and ranged from 3 to 8 days.

### Water column incubations

Light and dark water column incubations were conducted at each sampling location and followed methods described in Larson et al. (2019). Light and dark incubations were performed to experimentally replicate diurnal patterns in light availability and capture variation in associated natural system functions (e.g. primary production and photodegradation). At each location, rivermouth water within photic zone was pumped into a clean, continuously stirred holding vessel and used to triple rinse and fill eight transparent (~ 70–80% UV transparent; Ferreyra et al., 1996) 1-L plastic bags with 900 mL of water (Whirl-Pak®, Madison, Wisconsin, USA). Each bag was then randomly assigned a light treatment (light or dark) and an incubation time (initial or final) ensuring there were two replicate bags for each treatment-time combination. For bags assigned to the dark treatment, a second opaque bag was used to cover the transparent bag and block visible light. Bags were then attached to a buoy in the rivermouth at about 1-m depth with the dark bags fastened approximately 10 cm below the light bags. The process of filling and deploying bags took less than 1 h and required bags to be temporarily (< 30 min) stored in a closed cooler with rivermouth water prior to deployment. All bags were deployed mid-morning (~ 9:00 AM). Rivermouth water used for water column incubations was also collected in 1-L Nalgene bottles to measure chlorophyll *a* concentration following methods described in Larson et al. (2016; 2019) (Table S1).

On retrieval (or on deployment for initial bags), bags were placed in a closed cooler on ice to slow biological activity. Water samples were collected immediately from each bag (< 1 h to collect samples from all bags), field filtered through a pre-rinsed 0.22-µm polycarbonate membrane and stored in dark glass vials at 4 °C prior to analysis for DOC concentration and DOM composition. Incubation durations differed slightly among sampling events and ranged from 6 to 12 h. Blank bags filled with MilliQ water were also deployed and retrieved during each event and used as an indicator of DOM contamination. No contamination issues were found with DOC measured in MilliQ blank bags at all timepoints having concentrations below method detection limits.

Stored filtrate collected during sediment and water column incubations was used to determine DOC concentration following persulfate acidification with a Shimadzu TOC-VWP Total Organic Carbon Analyzer where analytical triplicates had coefficients of variation (CV) of less than 0.02. One analytical replicate water sample from the start and end of each individual sediment and water column incubation was further analyzed for DOM composition through spectrofluorometric characteristics. A Varian Cary 50 Bio UV-Visible spectrophotometer was used to measure DOM absorbance from 800 to 230 nm and a Varian Cary Eclipse Fluorometer was used to conduct visible 3D excitation-emission matrix (EEM) scans on each water sample. EEM scans were made at 0.25 nm s^− 1^ with 5 nm band widths over a 600–270 emission (2 nm interval) and 500–230 nm (5 nm interval) excitation range (Murphy et al., 2010; Williams et al., 2010). Instrument bias, inner filter effects, and blank Milli-Q EEM scans were used to correct sample EEMs (Cory et al., 2010; Murphy et al., 2010), and the area under the Raman scatter peak (350 nm excitation) from Milli-Q blank scans was used to standardize sample EEM fluorescence to Raman Units (RU). DOM composition was described by five common indices (Table S2): (1) UV absorbance at 254 nm (SUVA_254_; Weishaar et al. 2003), (2) a spectral slope ratio (S_R_; Helms et al. 2008), (3) the beta-alpha ratio or freshness index (β:α; Parlanti et al. 2000; Wilson and Xenopoulos 2009), (4) the fluorescence index (FI; Cory et al. 2010; McKnight et al. 2001), and (5) a modified humification index (HIX; Ohno 2002; Zsolnay et al. 1999;).

Furthermore, corrected EEM scans were fitted to an existing parallel factor analysis (PARAFAC) model suited to our study area (Williams et al. 2013; 2016). PARAFAC components were estimated using the DOMFluor Toolbox and the intensity (Fmax) of each component in a sample was used as a proxy of concentration for each compound in the PARAFAC model. Seven PARAFAC components were extracted and described as ubiquitous humic-like (C1), terrestrial humic-like (C2-C3), soil fulvic-like (C4), microbial humic-like (C5-C6), and microbial protein-like (C7) DOM properties (Williams et al., 2013; 2016; Table S2). Similar PARAFAC components are often correlated (Williams et al., 2013; 2016), thus Fmax values were summed to generate a composite terrestrial humic-like component (C_TH_ = C1 + C2 + C3) and microbial humic-like component (C_MH_ = C5 + C6). For clarity, the two remaining components were renamed based on their DOM properties: C_F_ (C4, soil fulvic-like) and C_P_ (C7, microbial protein-like). Select replicates of both absorbance and fluorescence scans used to quantify instrumental error had a mean CV of less than 0.09 across all measured DOM indices.

Total dissolved nitrogen (TDN) and phosphorus (TDP) concentrations were also measured during incubation experiments and used to calculate nutrient uptake rate in two previous studies (Larson et al. 2019; Pearce et al. 2021). We used the initial TDN and TDP concentrations from each water column incubation to determine the influence of dissolved nutrients on DOC and DOM transformation in the water column (Table S1).

### Data analysis

Initial (i.e. day 0) and final samples collected from sediment and water column incubations at each site were used to estimate net changes in DOC and DOM composition per unit time (Δ = [final – initial] / time). Net changes from individual incubations were then scaled to the entirety of the Fox rivermouth to account for the relative contributions of benthic and water column processes at the rivermouth scale. Non-scaled volumetric and areal rates were reported in the supplementary material (Table S3, Figures S1-S3). For sediment incubations, water samples collected after 1 day were most common and used as the final sample for all incubation experiment calculations apart from the April sampling events in 2016 where only samples collected after 8 days were available. Sediment DOC transformation rates were converted to units of mg C per day relative to the three-dimensional surface area calculated from the bathymetry of the Fox rivermouth (4,837,325.7 m^2^). For water column incubations, DOC processing rates were also converted to units of mg C per day, but rates were corrected to integrate biological activity occurring in the photic zone of the Fox rivermouth. Photic zone depth (i.e. PAR < 25 µmol m^− 2^ s^− 1^) for each sampling event was estimated from the light attenuation coefficient calculated from field measurements of PAR collected at and 1.54 m below the water surface (Wetzel and Likens 2000). The volume of water corresponding to the mean photic zone depth of 1.76 m across the entirety of the Fox rivermouth (4,472,389.2 m^3^) was used to scale water column DOC processing rates. In addition, the mean duration of daylight (April to September; ~ 14 h) was used to scale all water column DOC processing rates to diurnal patterns in light availability. A mean daily DOC budget was then estimated from sediment, light, and dark water column DOC processing rates by summing the means and posterior probability distributions from each set of observations. Normal posterior probability distributions for sediment and water column transformation rates were computed using the BEST package in R (Kruschke and Meredith 2021) and were informed with broad priors based on the mean and upper 90% confidence interval of the standard deviation from all associated observations. The percentage of the posterior probability distribution surrounding zero was computed to provide context on the directionality of processing rates. Instances where > 90% of the distribution did not overlap zero were considered to reflect strong unidirectional changes in DOC. Credible intervals (80%) were reported to express critical values for a one-tailed 90% probability distribution.

Because the other DOM properties were measured as fluorescence-based indices, DOM compositional change per day was calculated from the absolute change in each property over the incubation period. The absolute change in DOM properties was further assumed to be constant throughout the photic zone of the water column. With this assumption, water column DOM compositional change was scaled to the rivermouth by correcting for the portion of the water column outside of the photic zone and then for the mean duration of daylight. Similarly, sediment-related changes in DOM composition were scaled to the total volume of water in the rivermouth (4,841,126.9 m^3^) based on the contribution of sediments to the absolute change in DOM properties in the volume of rivermouth water directly above the sediment surface (4,837,325.7 m^2^ × 0.09 m). Means and posterior probability distributions were then calculated for each distribution of observations and used to determine if net DOM transformations were unidirectional based on their percentage overlap with zero whereby less than 10% overlap indicated strong unidirectional changes. The means and posterior probability distributions for each DOM property were then summed to determine the mean net daily change in DOM in the rivermouth. As optical properties and PARAFAC components differed in units, observations and computed values were centered to zero and scaled by the overall standard deviation from all incubation experiments for visualization purposes.

Contingency tables were used to determine which DOM properties changed alongside the bulk DOC concentration. DOC fluxes were converted to a categorical variable with levels of positive (i.e. increased DOC, +) and negative (i.e. decreased DOC, –). Likewise, a categorical variable was used to describe the change in DOM optical properties over the incubations (increase, +; or decrease, –). Bayes factors were used to evaluate evidence against the null hypothesis that the direction of change in DOM optical properties occurred independently of the direction of DOC fluxes. Contingency tables were evaluated in R with the BayesFactor package using a prior concentration of 1 and Poisson sample type (Morey et al. 2018). Bayesian factors are interpreted as the ratio of the likelihood of the alternative hypothesis over the likelihood of the null hypothesis. Support for the alternate hypothesis is typically considered strong when bayes factors are greater than 10 (Wasserman 2000).

Water column DOC and associated DOM composition change were further investigated to examine the influence of dissolved nutrients and chlorophyll *a* on water column processes. Partial regression analyses were performed between dependent (volumetric DOC flux rates; mg C L^− 1^ day^− 1^, and absolute DOM compositional change; Δ day^− 1^) and independent (initial TDN, TDP, and chlorophyll *a*) variables using Bayesian generalized linear models. Partial effects between independent and dependent variables were evaluated based on model regression coefficients and considered strong if 90% of posterior probability distribution of regression coefficients did not overlap zero. Bayesian generalized linear models were computed in R with the rstanarm package (Gabry et al. 2020).

## Results

### Rivermouth DOC concentration and DOM composition

Initial DOC concentrations in water column incubations and the overlying water in sediment incubations ranged from 6.62 to 13.99 mg L^− 1^ with a mean of 8.60 mg L^− 1^ and 9.04 mg L^− 1^ in water column and sediment incubations, respectively (Table 1). DOM composition was indicative of largely terrestrial-like, humic-like, and high molecular weight DOM as shown by higher values of SUVA_254_ (~ 2.8) and HIX (~ 7.6), and lower values of S_R_ (~ 1.0), β:α (~ 0.7), and FI (~ 1.4). PARAFAC components C_TH_ and C_MH_ made up the largest fraction of the DOM pool with C_F_ and C_P_ accounting for a lower proportion. Except for water column C_P_, initial DOC concentrations and DOM composition were largely invariable at the start of sediment and water column incubation experiments (CV < 0.5).

**Table 1:** Initial DOC concentrations (mg L^-1^) and DOM composition for sediment and water column incubation experiments. PARAFAC components are expressed in units of fluorescence (Fmax) and represent terrestrial humic (TH), soil fulvic (F), microbial humic (MH), and microbial protein (P) like DOM.

	DOC	SUVA_254_	S_R_	HIX	β:α	FI	C_TH_	C_F_	C_MH_	C_P_
Water column										
Mean	8.60	2.87	1.01	7.22	0.74	1.40	3.28	0.24	0.94	0.62
St. Dev.	1.03	0.36	0.05	2.43	0.03	0.05	0.46	0.03	0.36	0.36
Median	8.79	2.79	1.01	7.59	0.73	1.40	3.13	0.24	0.99	0.50
Min.	6.62	2.25	0.91	1.97	0.68	1.32	2.76	0.17	0.02	0.30
Max.	10.53	3.62	1.10	11.36	0.81	1.50	4.29	0.30	1.48	1.56
CV	0.12	0.13	0.05	0.34	0.04	0.04	0.14	0.13	0.38	0.58
Sediment										
Mean	9.04	2.72	0.98	8.18	0.75	1.41	3.15	0.25	1.27	0.44
St. Dev.	2.18	0.58	0.06	1.51	0.04	0.07	0.24	0.03	0.14	0.09
Median	8.29	2.81	0.98	7.51	0.74	1.40	3.14	0.24	1.28	0.45
Min.	7.19	1.66	0.91	6.54	0.69	1.29	2.64	0.21	1.01	0.29
Max.	13.99	3.32	1.10	11.56	0.81	1.52	3.45	0.32	1.45	0.58
CV	0.24	0.21	0.06	0.18	0.05	0.05	0.08	0.12	0.11	0.20

### Rivermouth DOC flux

The change in DOC concentrations ranged from − 3.72 to 3.06 mg L^− 1^ day^− 1^ and − 3.42 to 2.58 mg L^− 1^ day^− 1^ in light and dark water column incubations, respectively, and from − 0.23 to 0.14 g m^− 2^ day^− 1^ during sediment incubations. No discernible patterns were observed in DOC processing rates among sampling sites or the time points that were sampled (Figures S1 to S3). DOC processing in light incubations were on average (± standard deviation) 0.79 ± 2.26 mg L^− 1^ day^− 1^ in April, − 0.99 ± 1.59 mg L^− 1^ day^− 1^ in June, 0.00 ± 0.27 mg L^− 1^ day^− 1^ in August, and − 0.68 ± 1.10 mg L^− 1^ day^− 1^ in September, which were paralleled by DOC processing in dark incubations of 0.47 ± 1.86 mg L^− 1^ day^− 1^, − 1.09 ± 2.15 mg L^− 1^ day^− 1^, 0.26 ± 0.67 mg L^− 1^ day^− 1^, and − 0.68 ± 1.10 mg L^− 1^ day^− 1^, respectively. DOC fluxes from sediment incubations conducted in April, June, August, and September were 0.01 ± 0.11 g m^− 2^ day^− 1^, − 0.11 ± 0.11 g m^− 2^ day^− 1^, 0.04 ± 0.02 g m^− 2^ day^− 1^, and 0.09 ± 0.06 g m^− 2^ day^− 1^. From upstream to downstream, DOC processing rates among sites were − 0.34 ± 1.99 mg L^− 1^ day^− 1^, − 0.18 ± 0.43 mg L^− 1^ day^− 1^, and − 0.38 ± 1.09 mg L^− 1^ day^− 1^ for light incubations; − 0.89 ± 1.72 mg L^− 1^ day^− 1^, -0.35 ± 0.30 mg L^− 1^ day^− 1^, and 0.18 ± 1.62 mg L^− 1^ day^− 1^ for dark incubations; and − 0.01 ± 0.14 g m^− 2^ day^− 1^, 0.00 ± 0.12 g m^− 2^ day^− 1^, 0.00 ± 0.15 g m^− 2^ day^− 1^, 0.02 ± 0.13 g m^− 2^ day^− 1^, and − 0.02 ± 0.10 g m^− 2^ day^− 1^ for sediment incubations.

**Fig. 2:**
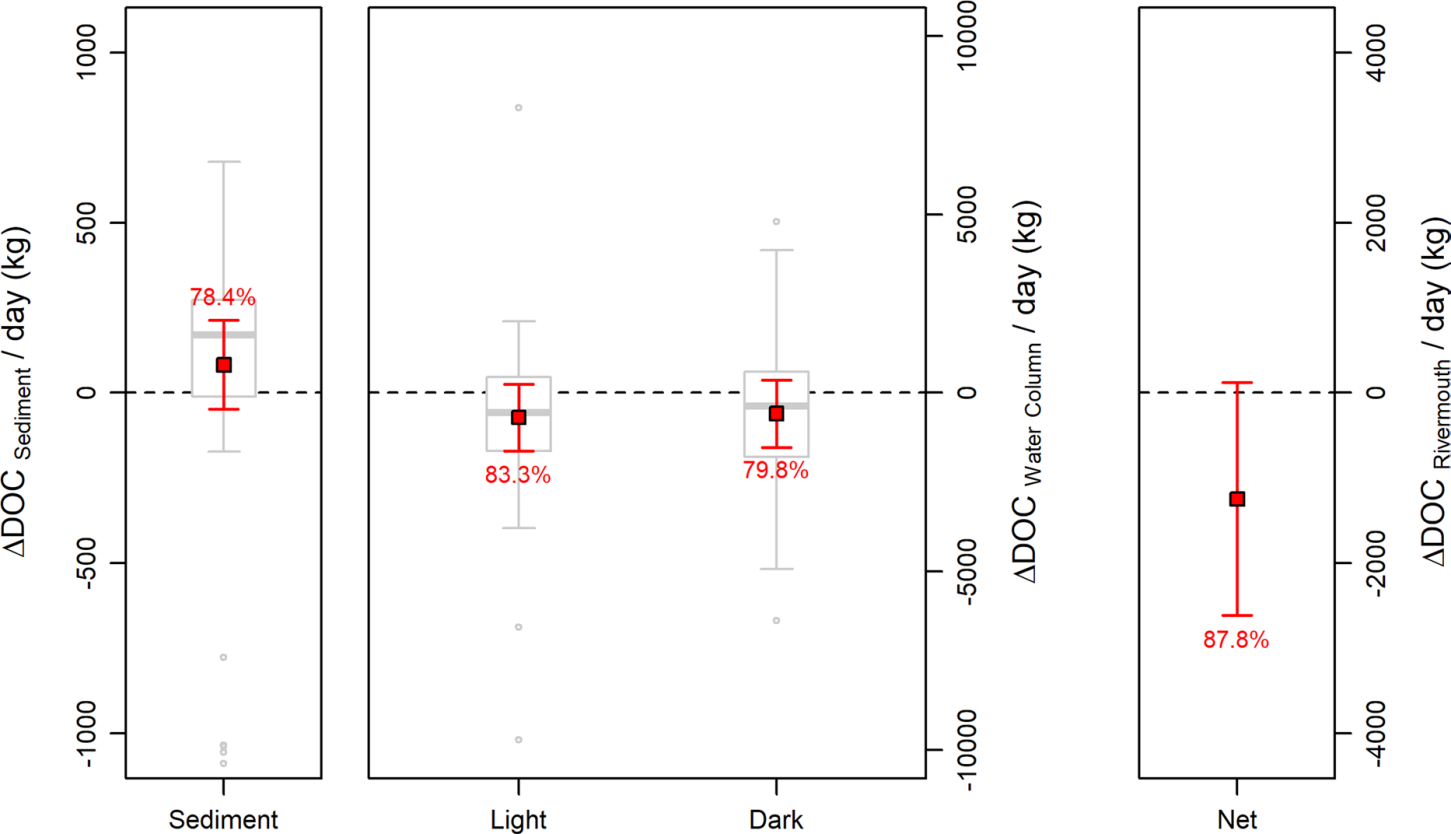
Summary of DOC fluxes from all sediment and water column (left) incubations, and net change in DOC in the Fox rivermouth (right). Negative values indicate decreases in DOC, and positive values indicate increases in DOC. Box plots show the median (line), quartiles (box extents), and 1.5 times the interquartile range (whiskers), and outliers (points) of the entire dataset. Red squares and error bars represent the mean and 80% credible interval of the posterior probability distribution with the percentage of the distribution outside of zero reported

When scaled to the entire rivermouth, sediment and water column processes were found to both increase and decrease the amount of DOC (Fig. 2); however, sediments largely released DOC (mean _[80% Credible Interval]_; 82.5 _[−39.5, 222.1]_ kg day^− 1^; 78.4% > 0) whereas DOC mineralization was more frequent in the water column with light (− 687.4 _[−1579.0, 290.5]_ kg day^− 1^; 83.3% < 0) and dark (− 582.4 _[−1494.3, 308.1]_ kg day^− 1^; 79.8% < 0) incubations having comparable DOC fluxes. On average, sediments contributed about 6% of the net change in DOC with the remaining change split evenly between processes occurring in light and dark conditions in the water column. The net DOC flux calculated from the posterior distributions of compiled sediment and water column rates was − 1187.3 _[−2492.3, 117.7]_ kg day^− 1^ and although the cumulative 90% credible interval of this net change overlapped zero, the Fox rivermouth largely resulted in a net decrease in DOC (87.8% < 0).

### Rivermouth DOM transformation

**Fig. 3:**
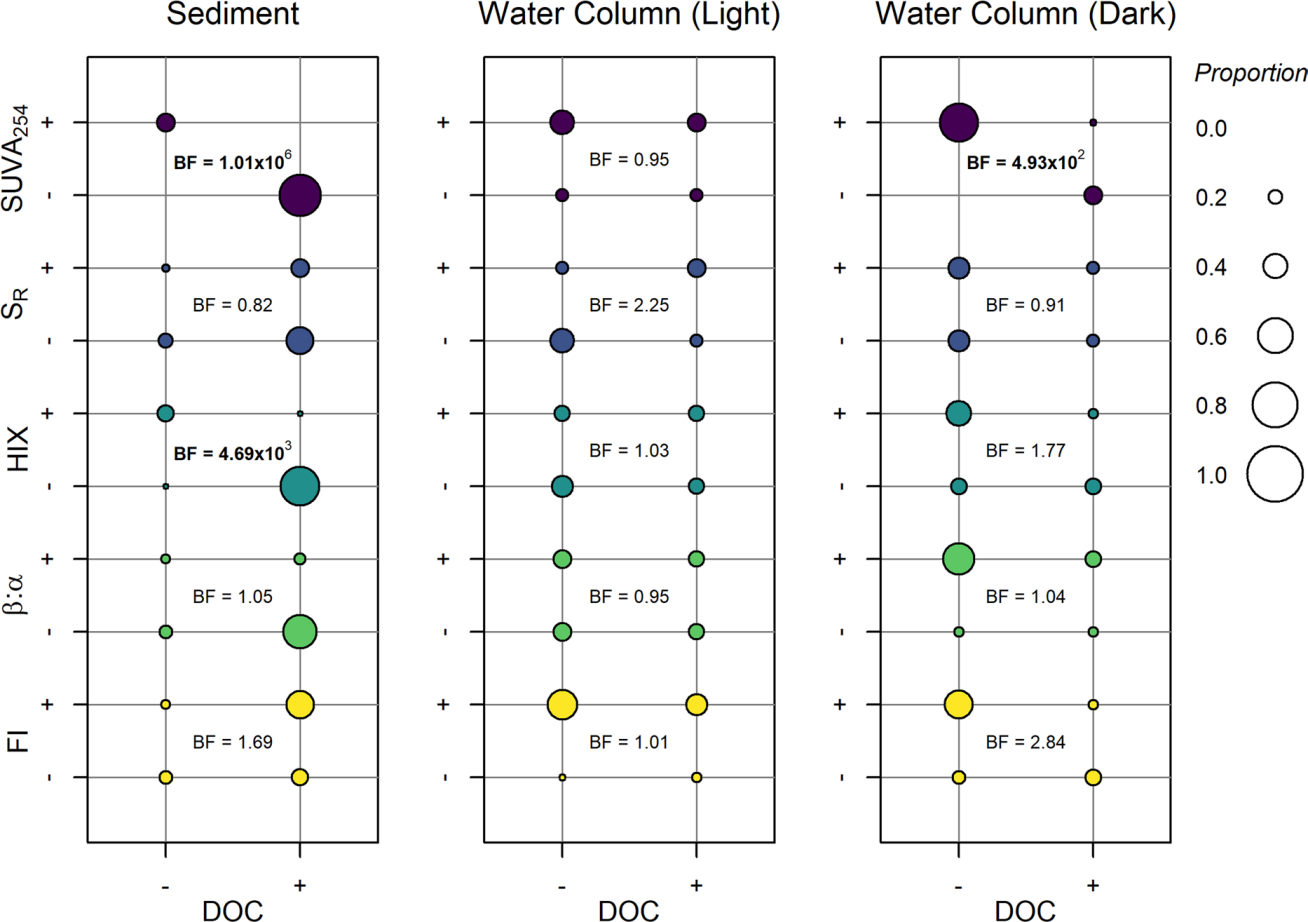
Contingency table between the change in DOM optical properties (increase, +; decrease, –) and DOC (increase, +; decrease, –) in sediment and water column incubation experiments. The size of each bubble indicates the proportion of observations for each condition. Bayes factors (BF) are reported whereby larger numbers indicate greater evidence against the null hypothesis of independence

**Fig. 4:**
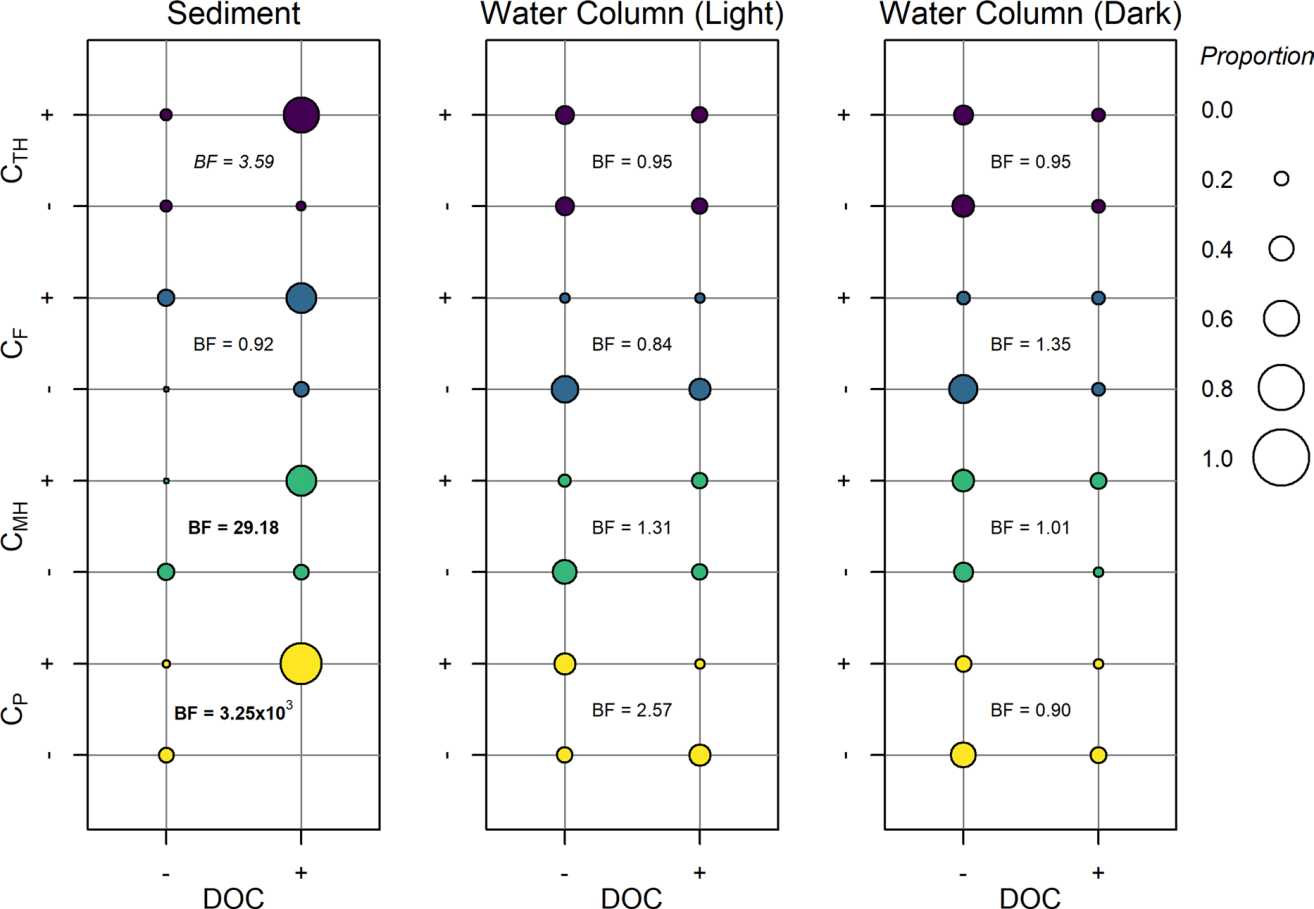
Contingency table between the change in PARAFAC components (increase, +; decrease, –) and DOC (increase, +; decrease, –) in sediment and water column incubation experiments. The size of each bubble indicates the proportion of observations for each condition. Bayes factors (BF) are reported whereby larger numbers indicate greater evidence against the null hypothesis of independence

Changes in DOC concentration were largely disconnected from those of DOM composition such that the direction of DOC fluxes were not associated with variation observed in several optical properties and PARAFAC components (Figs. 3 and 4). However, bulk DOC release from sediments was related to changes seen in SUVA_254_ and HIX with DOC release resulting in lower values of both optical properties in the overlying water column (Fig. 3). Similarly, sediment DOC release to the water column increased the amount of DOM associated with C_MH_ and C_P_ (Fig. 4). Within the water column, processing of DOC was only associated with changes in SUVA_254_ during dark incubation experiments; and in contrast to the sediments, DOC fluxes measured in dark water column incubations had an inverse relationship with SUVA_254_ where a decrease in DOC resulted in the increase of humic-like aromatic DOM in the water column (Fig. 3).

**Fig. 5:**
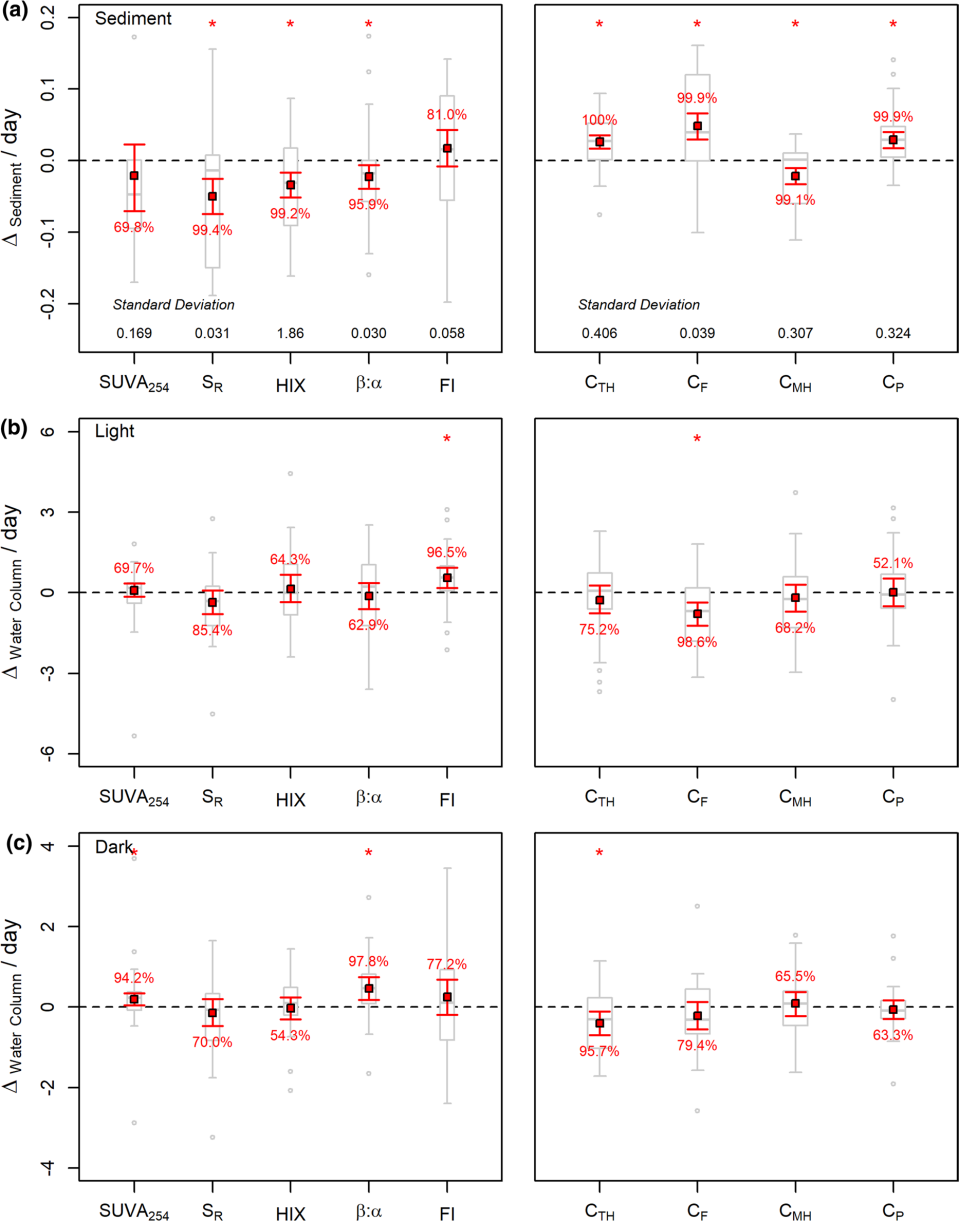
Summary of the change in DOM composition over all sediment (**a**) and light (**b**) and dark (**c**) water column incubation experiments at the rivermouth scale. DOM properties (left) and PARAFAC components (right) were centered to zero and scaled by the global standard deviation for visual comparison. Box plots show the median (line), quartiles (box extents), and 1.5 times the interquartile range (whiskers), and outliers (points) of the entire dataset. Negative values indicate a decrease and positive values indicate an increase in optical properties over the incubation. Red squares and error bars represent the mean and 80% credible interval of the posterior probability distribution with the percentage of the distribution outside of zero reported. Asterisk indicates strong unidirectionality in mean values signified by greater than 90% of the posterior probability distribution outside of zero

**Fig. 6:**
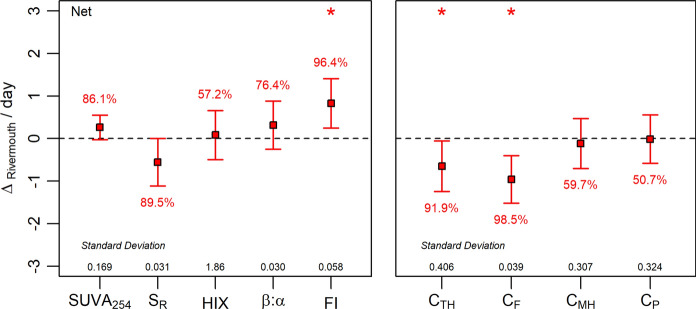
Summary of net changes in DOM composition at the rivermouth scale. DOM properties (left) and PARAFAC components are centered to zero and scaled by standard deviation for visualization. Positive and negative values indicate an increase or a decrease in DOM properties, respectively. Red squares and error bars represent the mean and 80% credible interval of the posterior probability distribution with the percentage of the distribution outside of zero reported. Asterisk indicates strong unidirectionality in mean values signified by greater than 90% of the posterior probability distribution outside of zero

At the rivermouth scale, light and dark water column incubations frequently resulted in larger changes in DOM optical properties and PARAFAC components than sediment incubations (Fig. 5). However, despite observing variation in the direction of DOM change for all measured optical properties and PARAFAC components, changes in sediment incubation DOM were more consistent than processes occurring in the water column. Sediment incubations resulted in a strong (> 90%) unidirectional decrease in SR, HIX, β:α, and C_MH_, and increase in C_TH_, C_F_, and C_P_ indicating the release of highly decomposed DOM and compounds associated with soil fulvic-like, terrestrial humic-like, and protein-like DOM properties. Similarly, light water column incubations revealed a strong unidirectional increase in FI, and decrease in C_F_; whereas dark water column incubations showed an increase in β:α, and decrease in C_TH_. Here, water column processing resulted in a decrease in soil fulvic-like and terrestrial humic-like DOM and an increase in microbial-like DOM. Overall, net DOM change calculated from the posterior distributions of compiled sediment and water column incubations indicated a strong unidirectional net increase in FI and net decrease in C_TH_ and C_F_ at the rivermouth scale; again, showing a decrease in terrestrial humic-like DOM and an increase in microbial-like DOM (Fig. 6). No discernible patterns were observed in DOM changes among sampling sites or the time points that were sampled (Figure S1 to S3).

### Influence of dissolved nutrients and chlorophyll

No partial effects of TDN, TDP, or chlorophyll *a* were observed on bulk DOC processing in light and dark conditions among water column incubation experiments (Table 2). Under light conditions, increased nutrients were associated with DOM compositional change where TDP was negatively associated with increases in C_TH_ and C_P_ and TDN with β:α indicating the greater mineralization/degradation of both terrestrial and microbially derived DOM. Chlorophyll *a* concentrations were also associated with the mineralization/degradation of C_TH_ and C_P_ but resulted in an increase of recently derived (β:α) and microbial humic-like DOM (C_MH_). More associations were observed in dark incubations, but the associations had less agreement between the different nutrients and indicators of DOM composition. However, like light incubations, TDP and chlorophyll *a* concentrations increased the mineralization/degradation of terrestrial humic-like (C_TH_) and microbial-like (C_P_, FI) DOM.

**Table 2:** Partial regression coefficients and 80% credible interval for Bayesian regression models between DOC transformation rates and the change in DOM composition, and TDN, TDP, and chlorophyll *a* (Chl *a*). Bolded values indicate partial effects that were supported by regression coefficients with 90% of the posterior probability distribution that did not overlap zero. Both dependent and independent variables were mean centered and scaled prior to analysis to standardize regression coefficients

	Light			Dark		
	TDN _| TDP + Chl *a*_	TDP _| TDN + Chl *a*_	Chl *a*_| TDN + TDP_	TDN _| TDP + Chl *a*_	TDP _| TDN + Chl *a*_	Chl *a*_| TDN + TDP_
DOC	− 0.02 _[−0.32, 0.33]_	− 0.20 _[−0.59, 0.19]_	0.02 _[−0.34, 0.38]_	0.05 _[−0.33, 0.42]_	− 0.05 _[−0.52, 0.42]_	0.11 _[−0.34, 0.57]_
SUVA_254_	− 0.31 _[−0.63, 0.01]_	0.25 _[−0.13, 0.63]_	0.04 _[−0.34, 0.42]_	**− 0.36**_**[−0.71, −0.02]**_	0.38 _[−0.01, 0.77]_	− 0.11 _[−0.49, 0.28]_
S_R_	− 0.18 _[−0.52, 0.13]_	0.30 _[−0.09, 0.67]_	− 0.02 _[−0.41, 0.36]_	**− 0.51**_**[−0.80, −0.23]**_	**0.55**_**[0.20, 0.91]**_	0.29 _[−0.06, 0.63]_
HIX	0.05 _[−0.29, 0.37]_	0.34 _[−0.06, 0.74]_	0.32 _[−0.07, 0.72]_	− 0.08 _[−0.42, 0.27]_	**0.48**_**[0.07, 0.93]**_	0.40 _[−0.03, 0.79]_
β:α	**− 0.35**_**[−0.64, −0.05]**_	0.17 _[−0.19, 0.53]_	**0.43**_**[0.07, 0.79]**_	**− 0.49**_**[−0.79, −0.19]**_	**0.61**_**[0.25, 0.97]**_	0.35 _[−0.01, 0.69]_
FI	− 0.05 _[−0.40, 0.30]_	0.06 _[−0.36, 0.47]_	0.00 _[−0.43, 0.41]_	− 0.10 _[−0.41, 0.24]_	**− 0.40**_**[−0.79, −0.02]**_	**− 0.66**_**[−1.02, −0.27]**_
C_TH_	0.10 _[−0.19, 0.38]_	**− 0.53**_**[−0.87, −0.19]**_	**− 0.63**_**[−0.98, −0.29]**_	**0.36**_**[0.04, 0.67]**_	**− 0.55**_**[−0.94, −0.16]**_	**− 0.39**_**[−0.74, −0.02]**_
C_F_	0.24 _[−0.08, 0.56]_	− 0.38 _[−0.77, 0.01]_	− 0.26 _[−0.64, 0.13]_	0.30 _[−0.06, 0.65]_	− 0.16 _[−0.61, 0.30]_	− 0.17 _[−0.61, 0.26]_
C_MH_	− 0.16 _[−0.48, 0.17]_	0.29 _[−0.09, 0.67]_	**0.44**_**[0.06, 0.81]**_	− 0.18 _[−0.57, 0.19]_	0.19 _[−0.25, 0.62]_	0.26 _[−0.17, 0.67]_
C_P_	0.02 _[−0.28, 0.34]_	**− 0.43**_**[−0.79, −0.05]**_	**− 0.52**_**[−0.88, −0.13]**_	0.14 _[−0.20, 0.49]_	**− 0.53**_**[−0.94, −0.12]**_	− 0.32 _[−0.72, 0.07]_

### Rivermouth DOC and DOM budget

**Fig. 7:**
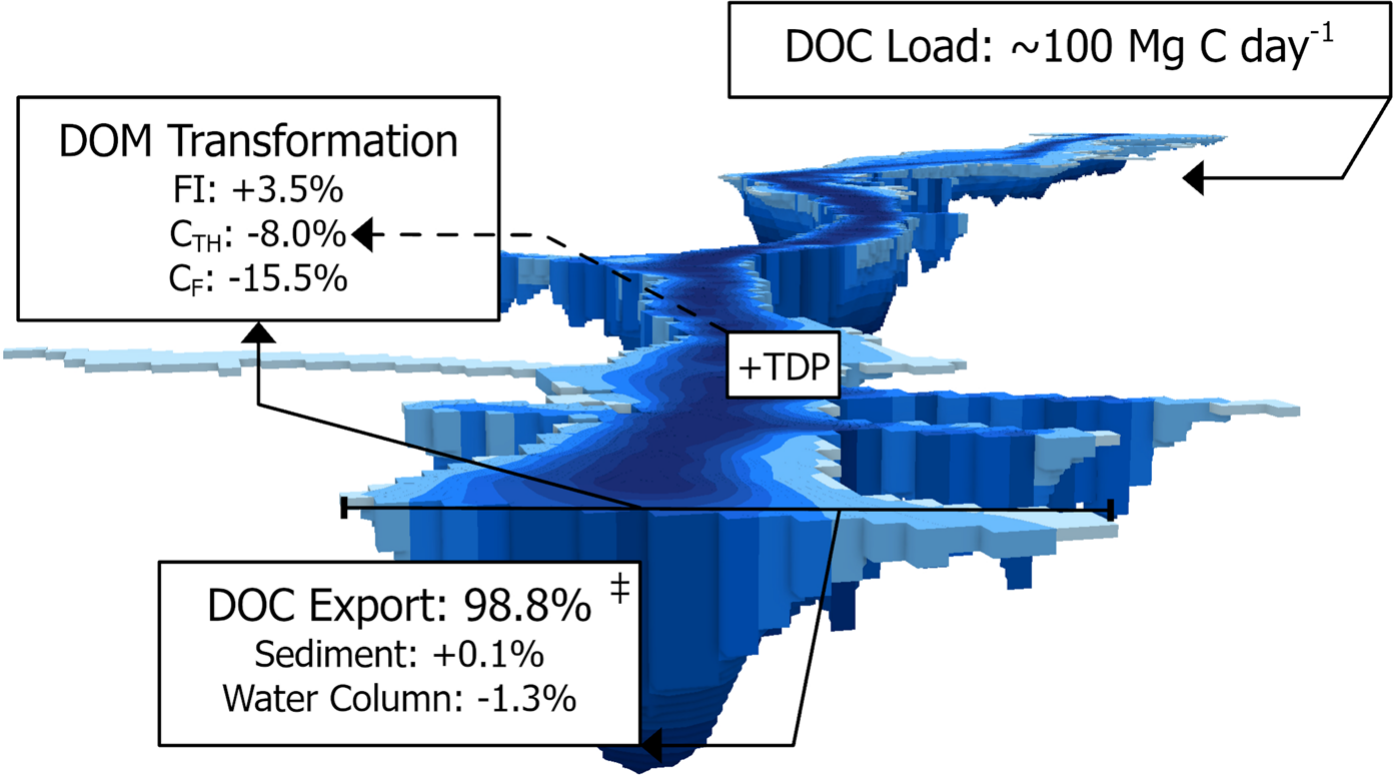
Summary of dissolved organic carbon (DOC) and dissolved organic matter (DOM) processing in the Fox rivermouth. Illustration not to scale (vertical exaggeration x100). ^‡^ Note, credible intervals overlap zero for DOC processing

Relative to the mean daily DOC load calculated from the mean initial DOC concentration (Table 1) and median daily discharge of the Fox River during our study period (April to September 2016–2017: ~135 m^3^ s^− 1^, USGS Station 040851385; U.S. Geological Survey, 2021), we estimated that the entirety of the Fox rivermouth, inclusive of the water column and sediment processes measured here, removes on average about 1.2% of the DOC that would be exported downstream per day (Fig. 7), but could remove as much as 2.4% or increase DOC export by 0.1% given the variability we observed. Likewise, relative to the mean initial water column DOM composition (Table 1), we found the Fox rivermouth to increase SUVA_254_, HIX, β:α, and FI, by 1.6%, 2.0%, 1.3%, and 3.5%, respectively; and decrease S_R_, C_TH_, C_F_, C_MH_, and C_P_ by 1.7%, 8.0%, 15.5%, 3.7%, and 0.6%, respectively, on average over a daily timescale. Yet, considering variation observed in DOM processing, strong unidirectional transformations at the rivermouth scale were limited to those that increased the freshness (FI) and decreased the terrestrial signature (C_TH_ and C_F_) of DOM (Fig. 7).

## Discussion

Biogeochemical processes that influence the movement of DOM along the aquatic continuum can have important implications in the local and global cycling of carbon (Xenopoulos et al. 2017), yet our understanding of spatial variation in the transformation of DOM as water moves across the landscape remains limited (Cory and Kling, 2018). Streams and lakes are widely considered active sites of carbon processing (Cole et al. 2007; Anas et al. 2015; Mineau et al. 2016) and here, we demonstrate that despite occupying a small fraction of the river network freshwater rivermouths can actively alter the concentration (DOC) and composition of DOM exported to receiving waterbodies (Fig. 7). The Fox rivermouth was a net sink of DOC, but we also observed it to be a net DOC source on some occasions (~ 12%). Water column processes rather than sediments were responsible for most of the bulk DOC mineralization with light and dark incubations having comparable rates. However, changes in DOM composition were not strongly coupled with changes in bulk DOC concentration as different forms of DOM were independently transformed through sediment and water column processes. We expected that nutrients would stimulate greater changes in DOC through increased phytoplankton and microbial activities, but we only found associations between dissolved nutrients and select DOM properties in the water column. Our study results indicate that water column processes (e.g. aquatic metabolism and photodegradation) can alter the composition of DOM and affect the quantity and biolability of carbon exported from rivermouth ecosystems but that it is inherently complex.

DOC was released through sediment processes, but DOC mineralization through water column processes was more influential and resulted in net DOC removal in the Fox rivermouth. DOC transformation rates in the Fox rivermouth converted to standard units (Table S3) varied considerably but were within the upper range of those reported in the water column of streams (Bernhardt and McDowell 2008; Mineau et al. 2016; Casas-Ruiz et al. 2017), large rivers (del Giorgio and Pace 2008), estuaries (Asmala et al. 2018), and in Lake Michigan (Biddanda and Cotner 2002); and were also comparable to DOC transformation rates reported in lake (Klump et al., 2009; Yang et al., 2014) and stream (Duan and Kaushal, 2013) sediments. Overall, 88% of our observations were indicative of net DOC removal in the Fox rivermouth, which is consistent with carbon budget of many other freshwater ecosystems (Tranvik et al. 2009; Anas et al. 2015; Mineau et al. 2016).

The net decrease in DOC within the Fox rivermouth indicates that more DOC was mineralized to carbon dioxide through microbial activities and/or photomineralization in the water column than was internally produced or released by sediments (Tranvik et al. 2009; Anas et al. 2015). Despite being mostly positive, sediment DOC fluxes were low compared to the large and mostly negative DOC fluxes that occurred within the water column. However, we did still observe instances where net increases in DOC were substantial within the Fox rivermouth. Phytoplankton can passively exude DOC as a by-product of photosynthesis and/or actively release DOC for various and poorly understood purposes (Marañón et al. 2004; Thornton 2014; Mühlenbruch et al. 2018). Phytoplankton exudation can be substantial, particularly during periods of high primary production (Baines and Pace 1991; Minor et al. 2006; Suksomjit et al. 2009) and is likely responsible for the positive DOC fluxes observed here. Moreover, given the magnitude and variability in water column DOC fluxes, it appears that aquatic metabolism (i.e. mineralization and exudation) was the dominant process controlling the carbon budget of the Fox rivermouth. Certainly, in our unconstrained incubation experiments photomineralization likely contributed to decreases in DOC under light conditions but compared to microbial processes, photomineralization rates are often low and less variable over time (Cory and Kling, 2018; Allesson et al., 2021; Maavara et al. 2021). Notably, the disparity between mean light and dark DOC mineralization aligns with estimates of photomineralization rates in large slow-moving rivers (Maavara et al. 2021), but further studies would be needed to better compartmentalize the rates of different ecosystem processes on rivermouth DOC fluxes.

DOC fluxes in benthic sediments of the Fox rivermouth were associated with the directional modification of DOM properties, but the direction of DOM transformation was mostly independent of DOC fluxes in the water column. Net DOC change in the water column was only associated with SUVA_254_ in dark incubations. Dark water column processes increased SUVA_254_ alongside DOC mineralization indicating the removal of less complex aliphatic DOM by heterotrophic microbes, but no discernible patterns in SUVA_254_ were observed in light incubations indicating that phytoplankton exudation or photodegradation may have decoupled the direction of DOM modification from that of DOC concentration. In contrast, sediments exchanged terrestrial derived and microbial-like DOM components, but largely resulted in a decrease in the humic and aromatic signature of DOM in the water column through the retention of more humic/aromatic DOM and/or the dilution from less humic/aromatic DOM. Peter et al. (2016) also showed decreases in SUVA_254_ and colored DOM (α420) over oxic sediment incubations from a boreal lake. However, Duan and Kaushal (2013) reported the opposite with increases in humic-like DOM to overlying from stream sediments. The release of DOC from sediments may be due to biotic (e.g. organic matter decomposition) or abiotic (e.g. dissolution and desorption) processes and be dependent on sediment composition (Hedges and Keil, 1995). It is possible that the retention of more complex DOM in sediments of the Fox rivermouth is due to physical processes as preferential mineralization by microbes should result in more complex refractory material remaining in the water (Duan and Kaushal, 2013). In addition, sediments often have a stronger potential to retain higher molecular weight DOM in mineral complexes (Jaffé et al. 2008; Xu et al. 2019; Xu et al. 2021). However, microbial metabolism can result in the production of complex DOM traditionally associated with allochthonous-like characteristics (Fox et al., 2017; Fox et al., 2021) which can make it difficult to interpret the diverse DOM transformations that occur in natural systems.

We found that changes in several DOM properties within sediment and water column incubation experiments were independent of DOC fluxes. DOM compositional changes were largely unidirectional, which indicates that some DOM transformations could be linked to more stable processes (i.e. retention/release, mineralization, or dilution). The consistent release of soil fulvic-like DOM (C_F_) and processes that led to the consistent decrease in S_R_ and β:α in sediment incubations ultimately resulted in more decomposed terrestrial derived DOM in the overlying water column regardless of the direction of DOC fluxes. In contrast, water column processes were found to consistently remove terrestrial derived DOM (C_TH_ and C_F_) but unidirectional decreases in this humic-like and fulvic-like DOM only occurred under dark and light conditions, respectively. Heterotrophic processes may therefore be more associated with the mineralization of terrestrial humic-like DOM whereas autotrophic processes may have contributed to the net removal of fulvic-like DOM, presumably through surface-DOM adsorption as fulvic acids readily adhere to phytoplankton cell walls (Vigneault et al., 2000; Sánchez-Marín and Beiras, 2011). Water column incubations were further found to increase the microbial composition of the DOM pool (β:α and FI), likely a result of phytoplankton exudation, but we did observe these changes in DOM composition under dark conditions indicating the potential for heterotrophic DOM release (Kawasaki and Benner, 2006) or continued autotrophic processing in the absence of light. Nevertheless, this result together with observations of positive DOC fluxes show that plankton-mediated DOM release may be a regular occurrence in the Fox rivermouth. Altogether, there was a net increase in microbial-like DOM characteristics (FI) and net decrease in terrestrial humic and fulvic-like components (C_TH_ and C_F_) in the Fox rivermouth (Fig. 7); again, largely regulated by water column processes. Therefore, rivermouths may behave similarly to lentic environments along the aquatic continuum and actively process DOM (Kling et al., 2000; Larson et al., 2007; Cory and Kling, 2018) resultantly increasing the microbial fraction exported to receiving waterbodies.

Contrary to our expectations and studies in other systems (e.g. Brailsford et al., 2021), dissolved nutrients (i.e. nitrogen and phosphorus) were not associated with changes in DOC concentration in the water column of the Fox rivermouth. A possible explanation for the apparent lack of a nutrient effect on water column DOC is the potential for nutrients to stimulate competing processes associated with both DOC mineralization and internal production (Mueller et al. 2016; Asmala et al. 2018; Livanou et al. 2019), thereby confounding net rate of change in experiments where autotrophic and heterotrophic processes are more balanced and occur together (Kadjeski et al. 2020). In addition, phytoplankton exude DOC through different cellular processes under both nutrient limited and replete conditions adding further complexity to nutrient-DOC relationships (Livanou et al. 2019; Thornton 2014). Nevertheless, we did observe some partial effects of dissolved nutrients on DOM compositional change, particularly modifications associated with DOM degradation. TDP was positively associated with the degradation of terrestrial humic-like (C_TH_), microbial protein-like (C_P_), and more recently derived DOM (β:α and FI) in both light and dark conditions thus explaining some of the variation observed in the transformation of these DOM properties. Partial effects of chlorophyll *a* concentrations on decreases in C_TH_ and C_P_ were also observed and appeared to reflect a priming effect (e.g. Ward et al. 2016) of more phytoplankton biomass on DOM processing. In addition, increased phytoplankton biomass as measured through chlorophyll *a* presumably increased exudation of microbial derived DOM (β:α and C_MH_) in the rivermouth (Polimene et al. 2017), but chlorophyll *a* was not associated with bulk DOC fluxes. Heterotrophic microbial biomass is likely another factor responsible for the variation observed in DOM transformation rates (Romaní et al. 2004; del Giorgio and Pace 2008; Brailsford et al., 2019); however, we did not quantify microbial biomass in our water column incubation experiments. Moreover, intermediary effects of environmental and physicochemical factors that regulate aquatic metabolism, in addition to other biogeochemically active components of rivermouths (e.g. macrophytes and animal excretion; Reitsema et al., 2018; Parr et al., 2020), may further affect and contribute to DOM transformations within these ecosystems. Additional research would therefore be beneficial to better quantify and fully understand variation in the movement and processing of carbon in these transitional ecosystems.

## Conclusion

River-to-lake transitional areas are biogeochemically active ecosystems that have the potential to interrupt the movement of solutes entering waterbodies from upstream surface waters (Larson et al. 2013; 2016). Here, we describe the transformation of DOM in the water column and sediments of a freshwater rivermouth (Green Bay, Lake Michigan) and show net DOC removal from the water column. On average, about 1187 kg C day^− 1^ was removed from the mouth of Fox River at the rivermouth scale. Based on compiled terrestrial export fluxes of DOC (Mineau et al. 2016; 23 to 1818 kg ha^− 1^ year^− 1^), the Fox rivermouth could remove up to 1% of the terrestrial DOC loaded from the watershed (16,650 km^2^) to the river network at an annual scale irrespective of temporal variation in biogeochemical processes, an estimate paralleled by the percentage (1.2%) of the mean DOC load that would be removed from the rivermouth per day (Fig. 7). This proportion is far larger than the fraction of the river network that the Fox rivermouth comprises (0.08% or 0.56% of Strahler order ≥ 4). However, the Fox rivermouth is intensively developed and receives a high nutrient load from agricultural and urban activities occurring in its watershed, likely augmenting rates of DOC mineralization and the microbial fraction of the DOM pool (Xenopoulos et al. 2021). We therefore expect DOM processing to vary spatially among rivermouths but given their unique physiography these transitional ecosystems may be hotspots for carbon processing. Temporal variation in carbon processing was also observed within the Fox rivermouth, and dissolved nutrients concentrations did explain some variation in the transformation of DOM properties, yet this variation was largely stochastic. Intra-annual variation in other environmental factors (e.g. light, temperature, and pollutants; Duan and Kaushal, 2013; Maavara et al. 2021; Brailsford et al., 2021) and changes in hydrology (i.e. water residence time and rivermouth volume; Soares et al., 2019) have the potential to alter the influence of rivermouth metabolism on carbon processing over shorter time scales and ultimately the export of DOC at the rivermouth scale; however, a higher resolution dataset would be required to start to elucidate these nuanced temporal patterns. Likewise, although multiple locations were sampled within the Fox rivermouth, our spatial coverage would need to be much greater to reliably interpolate and scale up any spatial heterogeneity in processing rates. It should therefore be recognized that by upscaling our rates with constant surface areas, volumes, and day lengths, our results simply reflect a first look of the prevalent carbon processing potential of the Fox rivermouth under base-flow conditions during the growing season. Thus, considerable uncertainties remain around spatial and temporal variation in the processing of DOM that warrants better understanding to reliably integrate the effects of ecosystem function and anthropogenic changes on the movement and processing of carbon along the aquatic continuum.

## Electronic supplementary material

Below is the link to the electronic supplementary material.

Supplementary Material 1

## Data Availability

The data and R script that support this research are available at 10.5066/P9Q1TI5E (Larson et al. 2022).

## References

[CR1] Allesson L, Koehler B, Thrane JE, Andersen T, Hessen DO (2021). The role of photomineralization for CO_2_ emissions in boreal lakes along a gradient of dissolved organic matter. Limnol Oceanogr.

[CR2] Anas MUM, Scott KA, Wissel B (2015). Carbon budgets of boreal lakes: state of knowledge, challenges, and implications. Environ Rev.

[CR3] Asmala E, Haraguchi L, Jakobsen HH (2018). Nutrient availability as major driver of phytoplankton-derived dissolved organic matter transformation in coastal environment. Biogeochemistry.

[CR4] Baines SB, Pace ML (1991). The production of dissolved organic matter by phytoplankton and its importance to bacteria: patterns across marine and freshwater systems. Limnol Oceanogr.

[CR5] Battin TJ, Kaplan LA, Findlay S (2008). Biophysical controls on organic carbon fluxes in fluvial networks. Nat Geosci.

[CR6] Battin TJ, Luyssaert S, Kaplan LA (2009). The boundless carbon cycle. Nat Geosci.

[CR7] Bernhardt ES, McDowell WH (2008). Twenty years apart: comparisons of DOM uptake during leaf leachate releases to Hubbard Brook Valley streams in 1979 versus 2000. J Geophys Res.

[CR8] Biddanda BA, Cotner JB (2002). Love handles in aquatic ecosystems: the role of dissolved organic carbon drawdown, resuspended sediments, and terrigenous inputs in the carbon balance of Lake Michigan. Ecosystems.

[CR9] Brailsford FL, Glanville HC, Golyshin PN (2019). Microbial uptake kinetics of dissolved organic carbon (DOC) compound groups from river water and sediments. Sci Rep.

[CR10] Brailsford FL, Glanville HC, Marshall MR (2021). Land cover and nutrient enrichment regulates low-molecular weight dissolved organic matter turnover in freshwater ecosystems. Limnol Oceanogr.

[CR11] Casas-Ruiz JP, Catalán N, Gómez-Gener L (2017). A tale of pipes and reactors: controls on the in-stream dynamics of dissolved organic matter in rivers. Limnol Oceanogr.

[CR12] Chen M, Hur J (2015). Pre-treatment, characteristics, and biogeochemical dynamics of dissolved organic matter in sediments: a review. Water Res.

[CR13] Cole JJ, Prairie YT, Caraco NF (2007). Plumbing the global carbon cycle: integrating inland waters into the terrestrial carbon budget. Ecosystems.

[CR14] Conroy JD, Kane DD, Quinlan EL, Edwards WJ, Culver DA (2017). Abiotic and biotic controls of phytoplankton biomass dynamics in a freshwater tributary, estuary, and large lake ecosystem: Sandusky Bay (Lake Erie) chemostat. Inland Waters.

[CR15] Cory RM, Kling GW (2018). Interactions between sunlight and microorganisms influence dissolved organic matter degradation along the aquatic continumn. Limnol Oceanogr Letters.

[CR16] Cory RM, Miller MP, McKnight DM (2010). Effect of instrument-specific response on the analysis of fulvic acid fluorescence spectra. Limnol Oceanogr Methods.

[CR17] Creed IF, McKnight DM, Pellerin BA (2015). The river as a chemostat: fresh perspectives on dissolved organic matter flowing down the river continuum. Can J Fish Aquat Sci.

[CR18] de Wit HA, Couture R-M, Jackson-Blake L (2018). Pipes or chimneys? For carbon cycling in small boreal lakes, precipitation matters most. Limnol Oceanogr Lett.

[CR19] del Giorgio PA, Pace ML (2008). Relative independence of organic carbon transport and processing in a large temperate river: the Hudson River as both pipe and reactor. Limnol Oceanogr.

[CR20] Drake TW, Raymond PA, Spencer RGM (2018). Terrestrial carbon inputs to inland water: a current synthesis of estimates and uncertainty. Limnol Oceanogr Lett.

[CR21] Duan SW, Kaushal SS (2013). Warming increases carbon and nutrient fluxes from sediments in streams across land use. Biogeosciences.

[CR22] Ferreyra GA, Demers S, del Giorgio P, Chanut J (1996). Physiological responses of natural plankton communities to ultraviolet-B radiation in Redberry Lake (Saskatchewan, Canada). Can J Fish Aquat Sci.

[CR23] Fisher TR, Harding LW, Stanley DW, Ward LG (1988). Phytoplankton, nutrients, and turbidity in the Chesapeake, Delaware, and Hudson estuaries. Estuar Coast Shelf Sci.

[CR24] Fong P, Zedler JB (2000). Sources, sinks, and fluxes of nutrients (N + P) in a small highly modified urban estuary in southern California. Urban Ecosyst.

[CR25] Fox BG, Thorn RMS, Anesio AM, Reynolds DM (2017). The in situ bacterial production of fluorescent organic matter; an investigation at a species level. Wat Res.

[CR26] Fox BG, Thorn RMS, Reynolds DM (2021). Laboratory in-situ production of autochthonous and allochthonous fluorescent organic matter by freshwater bacteria. Microorganisms.

[CR78] Gabry J, Ali I, Brilleman S (2020) rstanarm: Bayesian Applied Regression Modeling via Stan. Version 2.21.1

[CR27] Hedges JI, Keil RG (1995). Sedimentary organic matter preservation: an assessment and speculative synthesis. Mar Chem.

[CR28] Helms JR, Stubbins A, Ritchie JD (2008). Absorption spectral slopes and slope ratios as indicators of molecular weight, source, and photobleaching of chromophoric dissolved organic matter. Limnol Oceanogr.

[CR29] Jaffé R, McKnight D, Maie N (2008). Spatial and temporal variations in DOM composition in ecosystems: the importance of long-term monitoring of optical properties. J Geophys Res Biogeosciences.

[CR30] Jin X, Wang S, Zhao H (2008). Effect of organic matter on DOM sorption on lake sediments. Environ Geol.

[CR31] Kadjeski M, Fasching C, Xenopoulos MA (2020). Synchronous biodegradability and production of dissolved organic matter in two streams of varying land use. Front Microbiol.

[CR32] Kawasaki N, Benner R (2006). Bacterial release of dissolved organic matter during cell growth and decline: molecular origin and composition. Limnol Oceanogr.

[CR33] Klarer DM, Milie DF (1989). Amelioration of storm-water quality by a freshwater estuary. Arch Hydrobiol.

[CR34] Kling GW, Kipphut GW, Miller MM, O’Brien WJ (2000). Integration of lakes and stream in a landscape perspective: the importance of material processing on spatial patterns and temporal coherence. Freshw Biol.

[CR35] Klump JV, Fitzgerald SA, Waples JT (2009). Benthic biogeochmical cycling, nutrient stoichiometry, and carbon and nitrogen mass balances in a eutrophic freshwater bay. Limnol Oceanogr.

[CR79] Kruschke JK, Meredith M (2021) BEST: Bayesian Estimation Supersedes the t-Test. Version 0.5.3 10.1037/a0029146 22774788

[CR36] Larson JH, Frost PC, Zheng Z (2007). Effects of upstream lakes on dissolved organic matter in streams. Limnol Oceanogr.

[CR80] Larson JH, Pearce NJT, Bailey SW, Trebitz AS, Steinman AD et al (2013) Great Lakes rivermouth ecosystems: Scientific synthesis and management implications. J Great Lakes Res 39:513–524. 10.1016/j.jglr.2013.06.002

[CR37] Larson JH, Frost PC, Vallazza JM (2016). Do rivermouths alter nutrient and seston delivery to the nearshore?. Freshw Biol.

[CR38] Larson JH, Evans MA, Fitzpatrick FA (2019). Water column nutrient processing rates in rivermouths of Green Bay (Lake Michigan). Biogeochemistry.

[CR39] Larson JH, James WF, Fitzpatrick FA (2020). Phosphorus, nitrogen and dissolved organic carbon fluxes from sediments in freshwater rivermouths entering Green Bay (Lake Michigan; USA). Biogeochemistry.

[CR40] Levin LA, Boesch DF, Covich A (2001). The function of marine critical transition zones and the importance of sediment biodiversity. Ecosystems.

[CR41] Livanou E, Lagaria A, Psarra S, Lika K (2019). A DEB-based approach of modeling dissolved organic matter release by phytoplankton. J Sea Res.

[CR81] Maavara T, Logozzo L, Stubbins A et al (2021) Does photomineralization of dissolved organics matter in temperate rivers? J Geophys Res Biogeosci 126. 10.1029/2021JG006402. e2021JG006402

[CR42] Marañón E, Cermeño P, Fernández E (2004). Significance and mechanisms of photosynthetic production of dissolved organic carbon in a coastal eutrophic ecosystem. Limnol Oceanogr.

[CR43] McKnight DM, Boyer EW, Westerhoff PK (2001). Spectrofluorometric characterization of dissolved organic matter for indication of precursor organic material and aromaticity. Limnol Oceanogr.

[CR44] Mineau MM, Wollheim WM, Buffam I (2016). Dissolved organic carbon uptake in streams: a review and assessment of reach-scale measurements. J Geophys Res Biogeosciences.

[CR45] Minor EC, Simjouw J-P, Mulholland MR (2006). Seasonal variations in dissolved organic carbon concentrations and characteristics in a shallow coastal bay. Mar Chem.

[CR82] Morey RD, Rouder JN, Jamil T et al (2018) BayesFactor: computation of Bayes factors for common designs. Version 0.9.12–4.2.

[CR46] Mueller B, den Haan J, Visser PM, Vermeij MJA, van Duyl FC (2016). Effect of light and nutrient availability on the release of dissolved organic carbon (DOC) by caribbean turf algae. Sci Rep.

[CR47] Mühlenbruch M, Grossart H-P, Eigemann F, Voss M (2018). Mini-review: Phytoplankton-derived polysaccharides in the marine environment and their interactions with heterotrophic bacteria. Environ Microbiol.

[CR48] Murphy KR, Butler KD, Spencer RGM (2010). Measurement of dissolved organic matter fluorescence in aquatic environments: an interlaboratory comparison. Environ Sci Technol.

[CR83] Pearce NJT, Larson JH, Evans MA, Frost PF, Xenopoulos MA (2021) Episodic nutrient addition affects water column nutrient processing rates in river-to-lake transitional zones. J Geophys Res Biogeosci 126. 10.1029/2021JG006374. e2021JG006374

[CR49] Ohno T (2002). Fluorescence inner-filtering correction for determining the humification index of dissolved organic matter. Environ Sci Technol.

[CR50] Omernik JM, Griffith GE (2014). Ecoregions of the conterminous United States: evolution of a hierarchical spatial framework. Environ Manage.

[CR51] Parlanti E, Wörz K, Geoffroy L, Lamotte M (2000). Dissolved organic matter fluorescence spectroscopy as a tool to estimate biological activity in a coastal zone submitted to anthropogenic inputs. Org Geochem.

[CR52] Parr TB, Vaughn CC, Gido KB (2020). Animal effects on dissolved organic carbon bioavailability in an algal controlled ecosystem. Freshw Biol.

[CR53] Peter S, Isidorova A, Sobek S (2016). Enhanced carbon loss from anoxic lake sediment through diffusion of dissolved organic carbon. J Geophys Res Biogeosciences.

[CR54] Polimene L, Sailley S, Clark D (2017). Biological or microbial carbon pump? The role of phytoplankton stoichiometry in ocean carbon sequestration. J Plankton Res.

[CR55] Reitsema RE, Meire P, Schoelynck J (2018). The future of freshwater macrophytes in a changing world: dissolved organic carbon quantity and quality and its interactions with macrophytes. Front Plant Sci.

[CR56] Romaní AM, Guasch H, Muñoz I (2004). Biofilm structure and function and possible implications for riverine DOC dynamics. Microb Ecol.

[CR57] Sánchez-Marín P, Beiras R (2011). Adsorption of different types of dissolved organic matter to marine phytoplankton and implications for phytoplankton growth and pb bioavailability. J Plankton Res.

[CR58] Skoog AC, Arias-Esquivel VA (2009). The effect of induced anoxia and reoxygenation on benthic fluxes of organic carbon, phosphate, iron, and manganese. Sci Total Environ.

[CR59] Soares ARA, Lapierre J, Selvam BP (2019). Controls on dissolved organic carbon bioreactivity in river systems. Sci Rep.

[CR60] Suksomjit M, Nagao S, Ichimi K (2009). Variation of dissolved organic matter and fluorescence characteristics before, during and after phytoplankton bloom. J Oceanogr.

[CR61] Tao S, Lin B, Liu X, Cao J (2000). Release kinetics of water soluble organic carbon (WSOC) from river sediment and wetland soil. Water Air Soil Pollut.

[CR62] Thornton DCO (2014). Dissolved organic matter (DOM) release by phytoplankton in the contemporary and future ocean. Eur J Phycol.

[CR63] Tranvik LJ, Downing JA, Cotner JB (2009). Lakes and reservoirs as regulators of carbon cycling and climate. Limnol Oceanogr.

[CR64] Vigneault B, Percot A, Lafleur M, Campbell PGC (2000). Permeability changes in model and phytoplankton membranes in the presence of aquatic humic substances. Environ Sci Technol.

[CR65] Ward ND, Bianchi TS, Sawakuchi HO (2016). The reactivity of plant-derived organic matter and the potential importance of priming effects along the lower Amazon River. J Geophys Res Biogeosci.

[CR66] Wasserman L (2000). Bayesian model selection and model averaging. J Math Psychol.

[CR67] Weishaar JL, Aiken GR, Bergamaschi BA (2003). Evaluation of specific ultraviolet absorbance as an indicator of the chemical composition and reactivity of dissolved organic carbon. Environ Sci Technol.

[CR68] Wetzel RG, Likens GE (2000). Light and temperature. Limnological analysis.

[CR69] Williams CJ, Yamashita Y, Wilson HF (2010). Unraveling the role of land use and microbial activity in shaping dissolved organic matter characteristics in stream ecosystems. Limnol Oceanogr.

[CR70] Williams CJ, Frost PC, Xenopoulos MA (2013). Beyond best management practices: pelagic biogeochemical dynamics in urban stormwater ponds. Ecol Appl.

[CR71] Williams CJ, Frost PC, Morales-Williams AM (2016). Human activities cause distinct dissolved organic matter composition across freshwater ecosystems. Glob Change Biol.

[CR72] Wilson HF, Xenopoulos MA (2009). Effects of agricultural land use on the composition of fluvial dissolved organic matter. Nat Geosci.

[CR73] Xenopoulos MA, Downing JA, Kumar MD (2017). Headwaters to oceans: ecological and biogeochemical contrasts across the aquatic continuum. Limnol Oceanogr.

[CR74] Xu H, Zou L, Guan D (2019). Molecular weight-dependent spectral and metal binding properties of sediment dissolved organic matter from different origins. Sci Total Environ.

[CR75] Xu Y, Li P, Zhang C, Wang P (2021). Spectral characteristics of dissolved organic matter in sediment pore water from Pearl River Estuary. Sci China Earth Sci.

[CR76] Yang L, Choi JH, Hur J (2014). Benthic flux of dissolved organic matter from lake sediment at different redox conditions and the possible effects of biogeochemical processes. Wat Res.

[CR77] Zsolnay A, Baigar E, Jimenez M (1999). Differentiating with fluorescence spectroscopy the sources of dissolved organic matter in soils subjected to drying. Chemosphere.

[CR84] U.S. Geological Survey, 2021, USGS 040851385 Fox River at oil tank depot at Green Bay, WI, in USGS water data for the Nation. U.S. Geological Survey National Water Information System database, accessed 2021-04-17, at https://doi.org/10.5066/F7P55KJN.[Site information directly accessible at https://waterdata.usgs.gov/nwis/dv?referred_module=sw&site_no=040851385.]

[CR85] Xenopoulos MA, Barnes RT, Boodoo KS et al (2021) How humans alter dissolved organic matter composition in freshwater: relevance for the Earth’s biogeochemistry. 10.1007/s10533-021-00753-3. Biogeochemistry

