# Supplementary material for: Dissolved organic matter transformations in a freshwater rivermouth
Source: Biogeochemistry. 2023 Mar 2;163(3):245–63. doi: 10.1007/s10533-022-01000-z (PMC10121504; doi:10.1007/s10533-022-01000-z)
Supplement: Supplementary file 1 — Supplementary Material 1 [file 10533_2022_1000_MOESM1_ESM.docx]

Supplementary Material for: **Dissolved organic matter transformations in a freshwater rivermouth**

Nolan J. T. Pearce, James H. Larson, Mary Anne Evans, Sean W. Bailey, Paul C. Frost, William F. James, and Marguerite A. Xenopoulos

**Contents of this file:**

Figure S1 to S3 and Table S1 to Table S3

**Additional Supplementary Material:**

Corresponding datasets (eight .csv files) and R script (one .R file) in a zip folder are available at <https://doi.org/10.5066/P9Q1TI5E>.


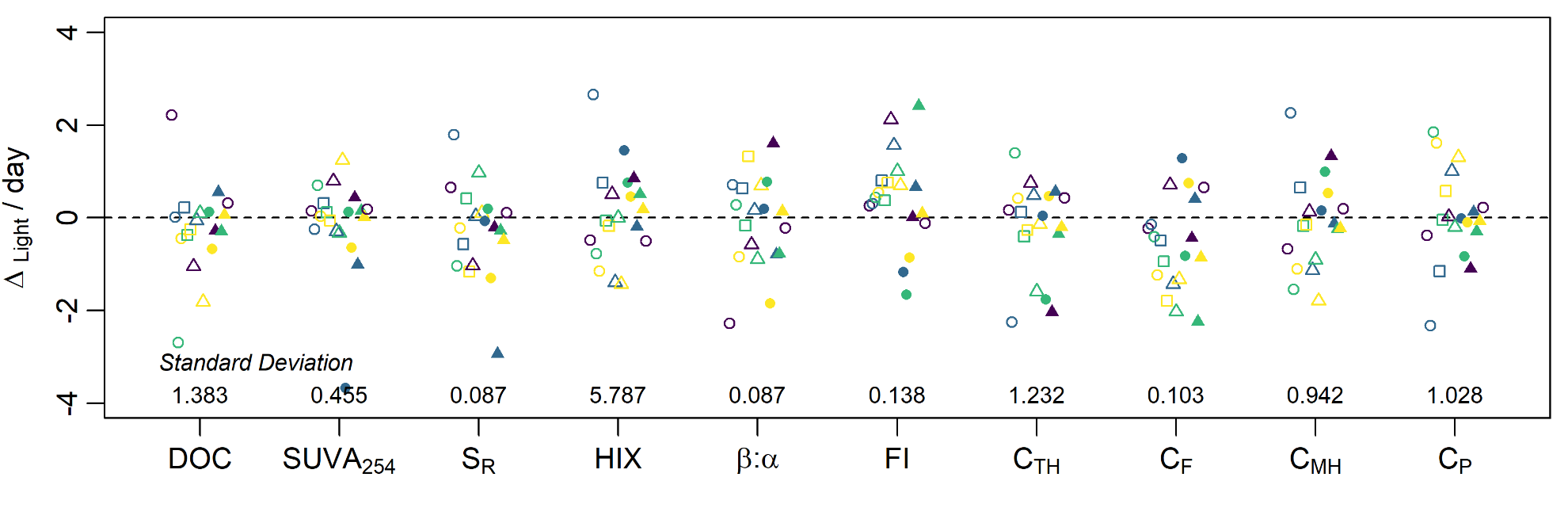


Figure S1 Summary of the change in dissolved organic carbon (DOC) concentration (mg L^-1^ day^-1^) and dissolved organic matter (DOM) composition (Δ day^-1^) over light water column incubation experiments by study site (circle = FX2; square = FX4; triangle = FX5) and time of sampling (purple = April; blue = August; green = June; yellow = September; open shape = 2016; closed shape = 2017). DOC and DOM transformation rates were centered to zero and scaled by standard deviation for visualization. Negative values indicate a decrease and positive values indicate an increase over the incubation. FX denotes the study site along the Fox rivermouth (see main text Figure 1). DOM indices include UV absorbance at 254 nm (SUVA_254_), a spectral slope ratio (S­_R_), a humification index (HIX), a beta-alpha freshness ratio (β:α), a fluorescence index (FI), a terrestrial humic-like component (C_TH_), a soil fluvic-like component (C_F_), a microbial humic-like component (C_MH_), and a microbial protein-like component (C_P_).


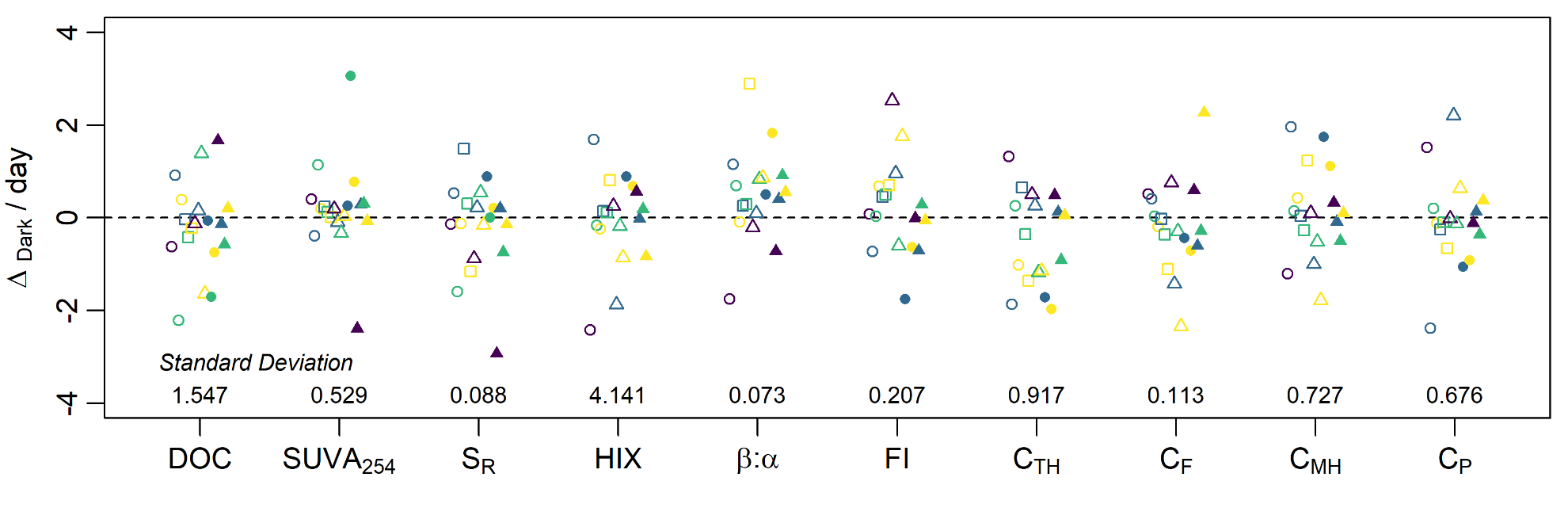


Figure S2 Summary of the change in dissolved organic carbon (DOC) concentration (mg L^-1^ day^-1^) and dissolved organic matter (DOM) composition (Δ day^-1^) over dark water column incubation experiments by study site (circle = FX2; square = FX4; triangle = FX5) and time of sampling (purple = April; blue = August; green = June; yellow = September; open shape = 2016; closed shape = 2017). DOC and DOM transformation rates were centered to zero and scaled by standard deviation for visualization. Negative values indicate a decrease and positive values indicate an increase over the incubation. FX denotes the study site along the Fox rivermouth (see main text Figure 1). DOM indices include UV absorbance at 254 nm (SUVA_254_), a spectral slope ratio (S­_R_), a humification index (HIX), a beta-alpha freshness ratio (β:α), a fluorescence index (FI), a terrestrial humic-like component (C_TH_), a soil fluvic-like component (C_F_), a microbial humic-like component (C_MH_), and a microbial protein-like component (C_P_).

**
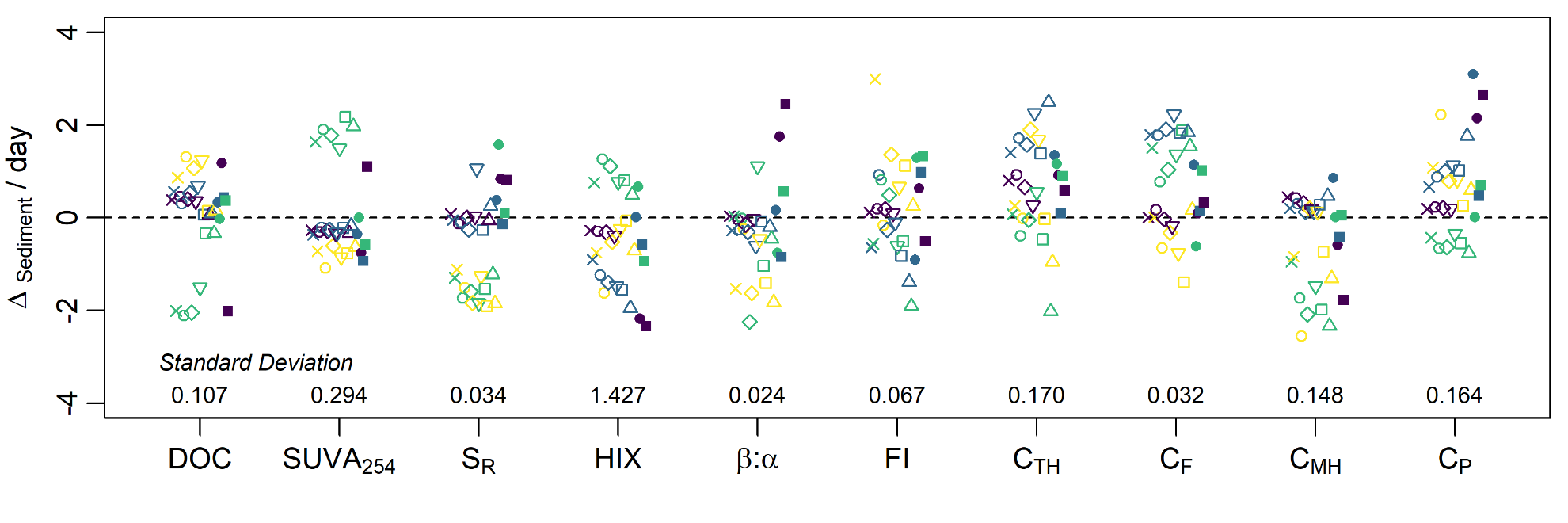
**

Figure S3 Summary of the change in dissolved organic carbon (DOC) concentration (g m^-2^ day^-1^) and dissolved organic matter (DOM) composition (Δ day^-1^) over sediment incubation experiments by study site (hollow circle = FX1; circle = FX2; diamond = FX3; square = FX4; triangle = FX4.5; inverse triangle = FX5) and time of sampling (purple = April; blue = August; green = June; yellow = September; open shape = 2016; closed shape = 2017). DOC and DOM transformation rates were centered to zero and scaled by standard deviation for visualization. Negative values indicate a decrease and positive values indicate an increase over the incubation. FX denotes the study site along the Fox rivermouth (see main text Figure 1). DOM indices include UV absorbance at 254 nm (SUVA_254_), a spectral slope ratio (S­_R_), a humification index (HIX), a beta-alpha freshness ratio (β:α), a fluorescence index (FI), a terrestrial humic-like component (C_TH_), a soil fluvic-like component (C_F_), a microbial humic-like component (C_MH_), and a microbial protein-like component (C_P_).

**Table S1** Descriptive summary of initial total dissolved phosphorus (TDP), total dissolved nitrogen (TDN), and chlorophyll a concentrations in water column incubation experiments (2016 – 2017).

|  | TDP (mg L^-1^) | TDN (mg L^-1^) | Chlorophyll *a* (µg L^-1^) |
| --- | --- | --- | --- |
| Mean | 0.024 | 0.97 | 27.5 |
| St. Dev. | 0.014 | 0.28 | 18.8 |
| Median | 0.021 | 0.94 | 21.4 |
| Min. | 0.004 | 0.57 | 6.8 |
| Max. | 0.057 | 1.42 | 79.7 |

Table S2 Description of dissolved organic matter (DOM) optical properties. Ex = excitation, Em = emission.

| DOM Optical Property | Description | Reference |
| --- | --- | --- |
| UV absorbance (254 nm) | Increased SUVA_254_ indicates greater carbon aromaticity or the number of aromatic rings per unit carbon. Strongly correlated and interchangeable with molar absorptivity (ε280). | Weishaar et al. (2003); Chin et al. (1994) |
| Spectral slope ratio (S_R_) | Increased S_R_ indicates lower molecular weight carbon and increased photobleaching. | Helms et al. (2008) |
| Beta-alpha ratio (β:α) | Increased β:α indicates a greater proportion of recently derived carbon (β) to highly decomposed carbon (α). Represents carbon “freshness”. | Parlanti et al. (2000); Wilson and Xenopoulos (2009) |
| Fluorescence index (FI) | Increased FI indicates microbial derived carbon (~1.9) and lower FI indicates terrestrial derived carbon (~ 1.4). | McKnight et al. (2001); Cory et al. (2010) |
| Humification index (HIX) | Increased HIX indicates more humic carbon components. | Zsolnay et al. (1999); Ohno (2002) |
| Component 1 (C1) | Ubiquitous humic-like DOM Ex: 260 (360); Em: 482 | Cory and McKnight (2005); Petrone et al. (2011) |
| Component 2 (C2) | Terrestrial humic-like DOM  Ex: 250 (310); Em: 420 (388) | Stedmon and Markager (2005) |
| Component 3 (C3) | Terrestrial humic-like DOM  Ex: 250; Em: 440 (468) | Stedmon and Markager (2005) |
| Component 4 (C4) | Terrestrial soil fluvic-like DOM  Ex: 285 (440); Em: 536 | Coble et al. (1990) |
| Component 5 (C5) | Microbial humic-like DOM  Ex: 360 (260); Em: 424 | Williams et al. (2013); Williams et al. (2010); Cory and Mcknight (2005) |
| Component 6 (C6) | Microbial humic-like DOM  Ex: 250 (285); Em: 386 | Williams et al. (2013); Williams et al. (2010); Cory and Mcknight (2005) |
| Component 7 (C7) | Microbial protein-like DOM  Ex: 280; Em: 342 (318) | Maie et al. (2008) |

Table S3 Summary of water column and sediment dissolved organic carbon (DOC) transformations reported in standardized unit. Values reported as the mean (median) ± standard deviation. Negative values indicate DOC removal. Values from this study were not corrected for duration of light per day.

|  |  | DOC Flux |
| --- | --- | --- |
| Fox rivermouth | Light | -583.5 (-372.0) ± 2435.7 mg m^-2^ day^-1^  -331.4 (-211.3) ± 1383.2 mg m^-3^ day^-1^  0.04 (0.02) ± 0.15 *k* day^-1^ |
|  | Dark | -568.8 (-354.8) ± 2723.8 mg m^-2^ day^-1^  -323.0 (-201.5) ± 1546.9 mg m^-3^ day^-1^  0.05 (0.03) ± 0.18 *k* day^-1^ |
|  | Sediment | 2.3 (35.2) ± 106.9 mg m^-2^ day^-1^ |
| Mineau et al. (2016) | Stream | 0.90 (0.01) ± 2.55 *k* day^-1^ |
| Bernhardt and McDowell (2008) | Stream | 0.0 to -8812.8 mg m^-2^ day^-1^ |
| Casa-Ruiz et al. 2017 | Stream | -189 to 164 mg m^-3^ hour^-1^ |
| del Giorgio and Pace (2008) | River | -5 to -29 mg m^-3^ day^-1^ |
| Asmala et al. 2018 | Estuary | -40.8 to -110.5 mg m^-3^ day^-1^ |
| Biddanda and Conter (2002) | Lake | -29 to -91 mg m^-2^ day^-1^ |
| Klump et al., 2009 | Sediments | 22 mg m^-2^ day^-1^ |
| Yang et al., 2014 | Sediments | 51 ± 100 mg m^-2^ day^-1^ |
| Duan and Kaushal, 2013 | Sediments | ~ 0.5 to ~ 5 mg m^-2^ day^-1^ |

References

Asmala E, Haraguchi L, Jakobsen HH, et al. (2018) Nutrient availability as major driver of phytoplankton-derived dissolved organic matter transformation in coastal environment. Biogeochemistry 137:93–104. https://doi.org/10.1007/s10533-017-0403-0

Bernhardt ES, McDowell WH (2008) Twenty years apart: Comparisons of DOM uptake during leaf leachate releases to Hubbard Brook Valley streams in 1979 versus 2000. J Geophys Res 113:G03032. https://doi.org/10.1029/2007JG000618

Biddanda BA, Cotner JB (2002) Love handles in aquatic ecosystems: The role of dissolved organic carbon drawdown, resuspended sediments, and terrigenous inputs in the carbon balance of Lake Michigan. Ecosystems 5:431–445. https://doi.org/10.1007/s10021-002-0163-z

Casas-Ruiz JP, Catalán N, Gómez-Gener L, et al. (2017) A tale of pipes and reactors: Controls on the in-stream dynamics of dissolved organic matter in rivers. Limnol Oceanogr 62:S85–S94. https://doi.org/10.1002/lno.10471

Cory RM, Miller MP, McKnight DM, et al. (2010) Effect of instrument-specific response on the analysis of fulvic acid fluorescence spectra. Limnol Oceanogr Methods 8:67–78. https://doi.org/10.4319/lom.2010.8.67

Cory RM, McKnight DM (2005) Fluorescence spectroscopy reveals ubiquitous presence of oxidised and reduced quinones in dissolved organic matter. Environ Sci Technol 39:8142–8149. https://doi.org/10.1021/ es0506962

del Giorgio PA, Pace ML (2008) Relative independence of organic carbon transport and processing in a large temperate river: The Hudson River as both pipe and reactor. Limnol Oceanogr 53:185–197. https://doi.org/10.4319/lo.2008.53.1.0185

Duan SW, Kaushal SS (2013) Warming increases carbon and nutrient fluxes from sediments in streams across land use. Biogeosciences 10:1193–1207. https://doi.org/10.5194/bg-10-1193-2013

Helms JR, Stubbins A, Ritchie JD, et al. (2008) Absorption spectral slopes and slope ratios as indicators of molecular weight, source, and photobleaching of chromophoric dissolved organic matter. Limnol Oceanogr 53:955–969. https://doi.org/10.4319/lo.2008.53.3.0955

Klump JV, Fitzgerald SA, Waples JT (2009) Benthic biogeochmical cycling, nutrient stoichiometry, and carbon and nitrogen mass balances in a eutrophic freshwater bay. Limnol Oceanogr 54(3): 692–712. https://doi.org/10.4319/lo.2009.54.3.0692

Maie N, Pisani O, Jaffé R (2008) Mangrove tannins in aquatic ecosystems: their fate and possible influence on dissolved organic carbon and nitrogen cycling. Limnol Oceanogr 53:160–171. https://doi.org/10.4319/lo.2008.53.1.0160

McKnight DM, Boyer EW, Westerhoff PK, et al. (2001) Spectrofluorometric characterization of dissolved organic matter for indication of precursor organic material and aromaticity. Limnol Oceanogr 46:38–48. https://doi.org/10.4319/lo.2001.46.1.0038

Mineau MM, Wollheim WM, Buffam I, et al. (2016) Dissolved organic carbon uptake in streams: A review and assessment of reach-scale measurements. J Geophys Res Biogeosciences 121:2019–2029. https://doi.org/10.1002/2015JG003204

Ohno T (2002) Fluorescence inner-filtering correction for determining the humification index of dissolved organic matter. Environ Sci Technol 36:742–746. https://doi.org/10.1021/es0155276

Parlanti E, Wörz K, Geoffroy L, Lamotte M (2000) Dissolved organic matter fluorescence spectroscopy as a tool to estimate biological activity in a coastal zone submitted to anthropogenic inputs. Org Geochem 31:1765–1781. https://doi.org/10.1016/S0146-6380(00)00124-8

Petrone JB, Fellman JB, Hood E, Donn MJ, Grierson PF (2011) The origin and function of dissolved organic matter in agro-urban coastal streams. J Geophys Res 116:G01028. https://doi.org/101029/2010JG001537

Stedmon CA, Markager S (2005) Resolving the variability in dissolved organic matter fluorescence in a temperate estuary and its catchment using PARAFAC analysis. Limnol Oceanogr 50:686–697. https://doi.org/10.4319/lo.2005.50.2.0686

Weishaar JL, Aiken GR, Bergamaschi BA, et al. (2003) Evaluation of specific ultraviolet absorbance as an indicator of the chemical composition and reactivity of dissolved organic carbon. Environ Sci Technol 37:4702–4708. https://doi.org/10.1021/es030360x

Williams CJ, Frost PC, Xenopoulos MA (2013) Beyond best management practices: Pelagic biogeochemical dynamics in urban stormwater ponds. Ecol Appl 23:1384–1395. https://doi.org/10.1890/12-0825.1

Williams CJ, Yamashita Y, Wilson HF, et al. (2010) Unraveling the role of land use and microbial activity in shaping dissolved organic matter characteristics in stream ecosystems. Limnol Oceanogr 55:1159–1171. https://doi.org/10.4319/lo.2010.55.3.1159

Wilson HF, Xenopoulos MA (2009) Effects of agricultural land use on the composition of fluvial dissolved organic matter. Nat Geosci 2:37–41. https://doi.org/10.1038/ngeo391

Yang L, Choi JH, Hur J (2014) Benthic flux of dissolved organic matter from lake sediment at different redox conditions and the possible effects of biogeochemical processes. Wat Res 61:97–107. https://doi.org/10.1016/j.watres.2014.05.009
